# Characterization of a novel androgen receptor (AR) coregulator RIPK1 and related chemicals that suppress AR-mediated prostate cancer growth via peptide and chemical screening

**DOI:** 10.18632/oncotarget.17843

**Published:** 2017-05-13

**Authors:** Cheng-Lung Hsu, Jai-Shin Liu, Ting-Wei Lin, Ying-Hsu Chang, Yung-Chia Kuo, An-Chi Lin, Huei-Ju Ting, See-Tong Pang, Li-Yu Lee, Wen-Lung Ma, Chun-Cheng Lin, Wen-Guey Wu

**Affiliations:** ^1^ Division of Hematology-Oncology, Departments of Internal Medicine, Chang Gung Memorial Hospital, Chang Gung University, Taoyuan 333, Taiwan; ^2^ Institute of Bioinformatics and Structural Biology, National Tsing Hua University, Hsinchu 300, Taiwan; ^3^ Department of Chemistry, National Tsing-Hua University, Hsinchu 300, Taiwan; ^4^ Division of Urology, Department of Surgery, Chang Gung Memorial Hospital, Chang Gung University, Taoyuan 333, Taiwan; ^5^ Department of Biological Science and Technology, National University of Tainan, Tainan 700, Taiwan; ^6^ Department of Pathology, Chang Gung Memorial Hospital, Chang Gung University, Taoyuan 333, Taiwan; ^7^ Sex Hormone Research Center, China Medical University Hospital, Taichung 104, Taiwan

**Keywords:** FxxLF, RIPK1, androgen receptor, prostate cancer, oxadiazole

## Abstract

Using bicalutamide-androgen receptor (AR) DNA binding domain-ligand binding domain as bait, we observed enrichment of FxxFY motif-containing peptides. Protein database searches revealed the presence of receptor-interacting protein kinase 1 (RIPK1) harboring one FxxFY motif. RIPK1 interacted directly with AR and suppressed AR transactivation in a dose-dependent manner. Domain mapping experiments showed that the FxxFY motif in RIPK1 is critical for interactions with AR and the death domain of RIPK1 plays a crucial role in its inhibitory effect on transactivation. In terms of tissue expression, RIPK1 levels were markedly higher in benign prostate hyperplasia and non-cancerous tissue regions relative to the tumor area. With the aid of computer modeling for screening of chemicals targeting activation function 2 (AF-2) of AR, we identified oxadiazole derivatives as good candidates and subsequently generated a small library of these compounds. A number of candidates could effectively suppress AR transactivation and AR-related functions *in vitro* and *in vivo* with tolerable toxicity via inhibiting AR-peptide, AR-coregulator and AR N-C interactions. Combination of these chemicals with antiandrogen had an additive suppressive effect on AR transcriptional activity. Our collective findings may pave the way in creating new strategies for the development and design of anti-AR drugs.

## INTRODUCTION

The androgen-androgen receptor (AR) pathway is important for physiological development and pathogenesis of diseases, such as prostate cancer [1]. AR is a transcription factor that belongs to the nuclear receptor superfamily [2, 3]. The transcriptional activity of AR is modulated by co-regulators, such as ARA70 and SHP [4]. Upon androgen binding to AR, the ligand binding domain (LBD) undergoes conformational changes and exposes its activation function 2 (AF-2) hydrophobic cleft to accommodate hydrophobic side-chains of peptides [5]. Traditionally, nuclear receptors bind the canonical LxxLL motif embedded within a short a-helix of co-regulators [6, 7]. AR prefers aromatic residue-rich FxxLF-like motifs [8]. The FxxLF-like motif exists in many AR coregulators and the AR N-terminus, and mediates AR-coregulator and AR N-C interactions [4, 8–11].

Anti-androgen withdrawal syndrome (AWS) describes the response to withdrawal of antiandrogen therapy [12, 13]. A number of hot spot mutations have been linked to treatment with individual anti-androgens, for instance, T877A with hydroxyflutamide (HF), W741(C/L) with bicalutamide (CDX) [14], and F876L with enzalutamide treatment [15]. The ligand specificity of T877A AR is lost, and as a result, the receptor can be activated by both androgens and anti-androgens [1]. Structural analyses have revealed that dihydrotestosterone (DHT)-wild type AR LBD and antiandrogen-mutant AR LBD display analogous binary structures and recruit similar FxxLF-like motif-containing peptides [16]. The tyrosine at position 5 of the Fxx(L/F)Y motif may provide an additional hydrogen bond for AR-peptide interactions compared to FxxLF motif peptides. Some antiandrogen-mutant AR complexes prefer FxxFY motif-containing peptides and could interact with BUD31 harboring a FxxFY motif that mediates AR-coregulator interactions [16].

In the current study, we demonstrated enrichment of FxxFY motif-containing peptides that interacted with CDX-W741L mutant AR LBD and identified RIPK1 harboring a FxxFY motif as a novel AR coregulator. We further assessed the utility of AF-2-targeting peptidomimetics in blocking AR N-C and AR-cofactor interactions as a potential novel anti-AR strategy for treatment of prostate cancer.

## RESULTS

**Figure 1:**
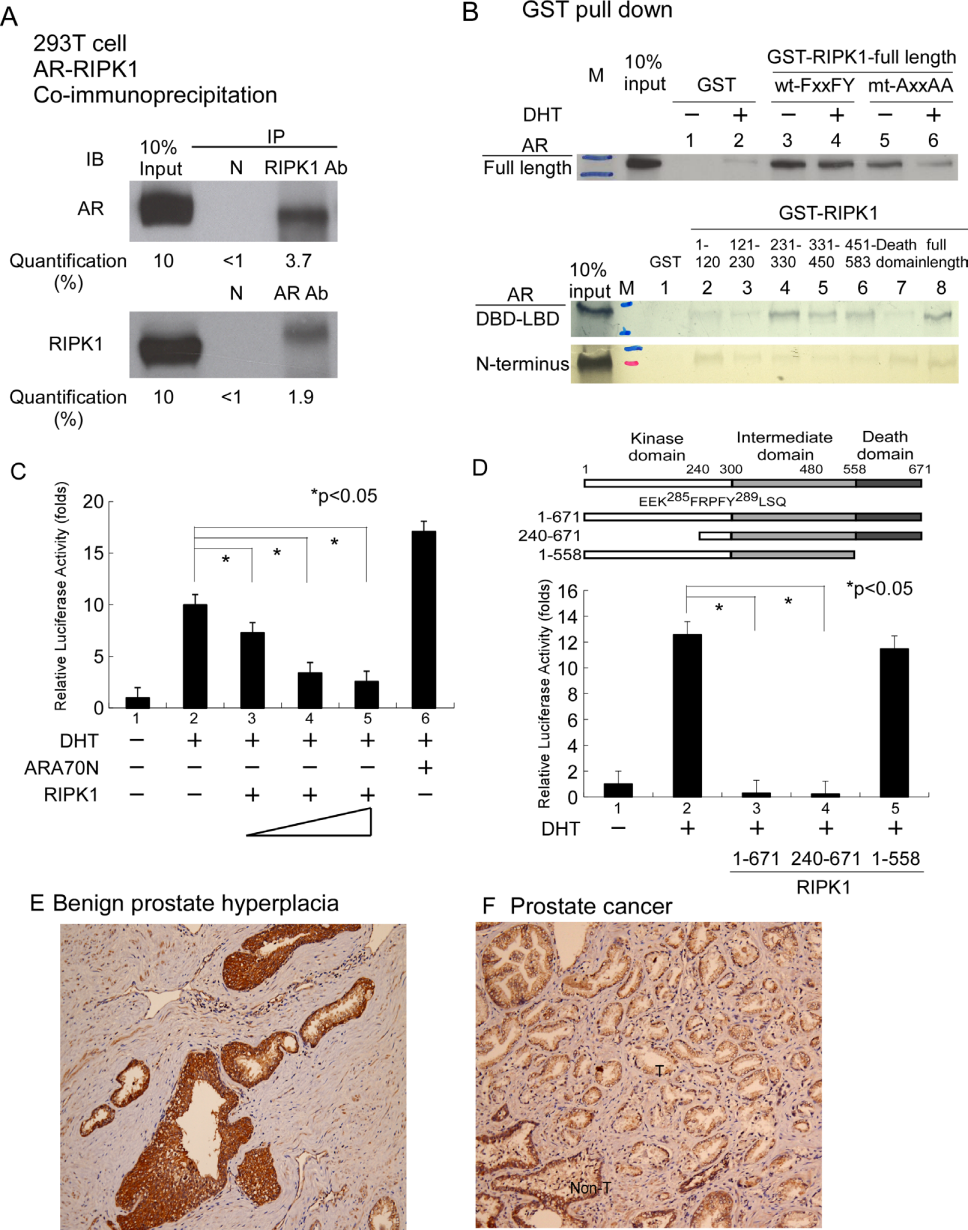
Associations between RIPK1 and AR (**A**) Co-IP of Flag-RIPK1 with Flag-AR in the 293T cell line. Extracts of 293T cells overexpressing 3xFlag-RIPK1 and 3xFlag-AR were treated with 1 mM DHT. IP was performed using anti-AR (C19) or anti-RIPK1 antibody or normal rabbit serum (negative control), followed by immunoblotting (IB) with antibodies against AR or RIPK1. (**B**) RIPK1 interacts with full-length AR and the N- and C-terminal regions of AR in GST pull-down assays. Mutation of the FxxFY motif to AxxAA in RIPK1 reduced interactions with AR. RIPK1 suppressed AR transactivation. (**C**) Transfection of PC-3 prostate cancer cells with AR and RIPK1. PC-3 cells in 24-well plates were co-transfected with 300 ng MMTV-LUC reporter plasmid and 0.5 ng SV40-*Renilla* luciferase plasmid, together with 100 ng pCMV-Flag-AR and 100, 300 or 500 ng p3xFLAG- RIPK1. The total plasmid DNA content was made up to 1 µg with pCMV. After 16 h, ethanol or 10 nM DHT was added and cells incubated for an additional 16 h. DHT was used as the AR ligand while ARA70N served as the positive control. Relative LUC activity was determined using the dual luciferase system. (**D)** RIPK1 functional domain mapping in relation to AR transactivation. PC-3 cells were transfected with pCMV-Flag-AR and RIPK1 expression plasmid, P3xFlag-RIPK1 full-length, P3xFlag-RIPK1-(240-671) or P3xflag-RIPK1-(1-558) plasmid, and cultured overnight. Ethanol or 10 nM DHT was added and cells incubated for an additional 16 h. Relative LUC activity was determined using the dual luciferase system. (**E**) RIPK1 is expressed in benign prostatic hyperplasia tissue, displaying strong positivity (3+, > 90%) in the gland area but weak (1+, 50%) staining in the background. (**F**) Human prostate cancer tissues were immunostained for RIPK1. “T” indicates the tumor area (right side) and “Non-T” the non-tumor area (left side). RIPK1 expression was weak (1+, 80%) in the cancer area but remained strong (3+, 70%) in the peri-cancer area. The figures are representative of three benign prostatic gland hyperplasia and cancer tissues.

Using the phage display technique, we observed enrichment of FxxFY motif-containing peptides in CDX-W741L AR LBD screening. Peptide-AR interactions were further validated with the mammalian two-hybrid assay. As shown in Table 1, the majority of screened peptides interacted with wild-type AR in the presence of DHT. The majority of these peptides additionally interacted with W741L-AR in the presence of bicalutamide. Protein database searches led to the identification of receptor-interacting protein kinase-1 (RIPK1) as a FxxFY motif-containing protein (^285^FRPFY^289^). RIPK1 is a kinase that plays a crucial role in inflammation and cell death [17]. We hypothesized that RIPK1 is an AR-interacting candidate protein and the FxxFY motif is involved in mediating interactions with AR. To examine this theory, we tested the short FxxFY-containing peptide in a mammalian two-hybrid assay. The results demonstrated strong interactions with AR, as shown in Table 1 (No. 13). Data from the co-immunoprecipitation assay showed that RIPK1 directly interacts with AR in 293T cells (pull-down of AR or RIPK1 and staining for both), as shown in Figure 1A. In interacting domain mapping, the FxxFY motif of RIPK1 influenced binding to AR in the GST pull-down assay, mediating AR interactions predominantly through DBD-LBD, as depicted in Figure 1B. RIPK1 significantly suppressed AR transactivation in a dose-dependent manner in the AR transcription assay (Figure 1C). RIPK1 contains an amino-terminal kinase domain, an intermediate domain and a carboxy-terminal death domain [17]. Functional domain mapping revealed that the death domain of RIPK1 influences its inhibitory effect on AR transactivation, although weak interactions of this domain with AR-DBD-LBD and AR N-terminus were detected (Figure 1C and 1D). We further examined tissue expression of RIPK1 in the prostate gland via immunohistochemical staining. As shown in Figure 1E and 1F, RIPK1 was enriched in benign prostate hyperplasia and non-tumor areas, but not the tumor area. In view of the collective data, we propose that RIPK1 is a bona fide AR coregulator.

**Table 1:** FxxFY motif was rich in bicalutamide (CDX)-W741L-AR associated peptide and most of screened peptides can interact with DHT-wt-AR and also with CDX-W741L-AR in MM2H assay

Peptide				wt-AR		W741L-AR	
	−1	+ 1 + 4 + 5	+6	Ethanol	DHT	Ethanol	CDX
Gal-4				1	1	1	1
1.C411-FY	NDTPVK	FAHFY	H	7	3	2	3
2.C414-FY	NPSSM	FEKFY	LR	10	2,653	2	532
3.C1-FY	SYT	FNQFY	YSTA	19	680	8	511
4.C412-FY	APSDTY	FQRFY	R	12	6	1	2
5.CA-10-FY	EHSM	FHSFY	VQG	12	277	4	14
6.C8-FY	GD	FKSFY	LATTW	17	484	2	86
7.C315-FY	APSLNR	FATFY	H	1	6	1	2
8.CA9-FY	SL	FTSFY	TGSGS	20	1,517	5	190
9.C4-FY	SPL	FSSFY	HART	4	340	1	114
10.CA23-FY	IQPSL	FAQFY	HP	46	213	32	4,118
11.CON-1	MYKP	HNHHQ	TSS	1	1	1	1
12.#3-18	NTNA	FSRLF	YPS	10	590	9	128
13.RIP-p	GIEEK	FRPFY	LSQLE	2	159	12	554

*Con-1, negative control.

**^#^3–18, positive control.

***RIP-p, short peptide of novel AR coregulator candidate.

DHT = 10 nM, CDX = 10 µM.

**Figure 2:**
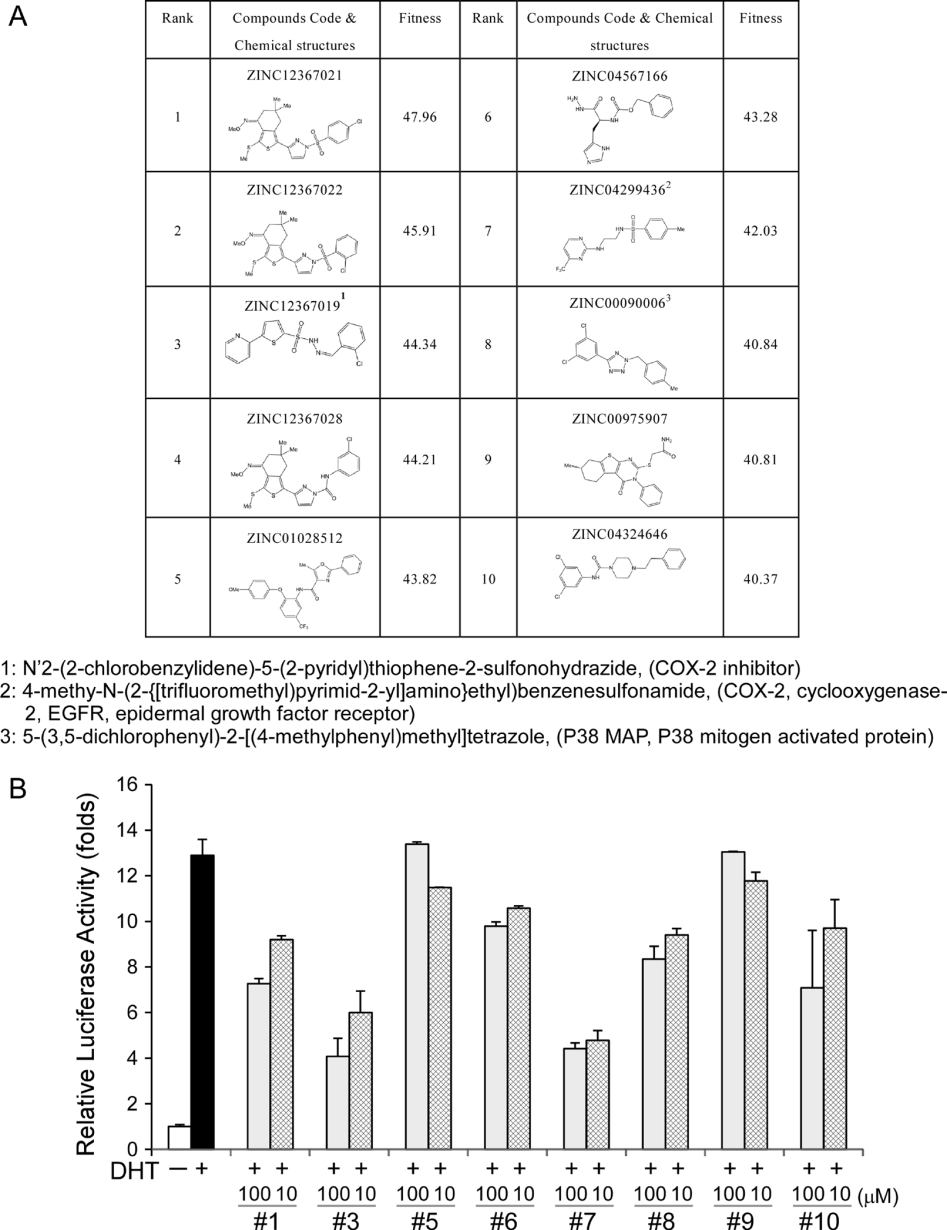
Computer modeling-screened candidate chemicals and their effects on AR transcriptional activity (**A**) Candidate chemicals identified from computer modeling and their structures. (**B**) Examination of the effects of 8 of the top 10 candidates on AR transcriptional activity.

Since our current findings, in addition to earlier literature reports, indicate that AF-2 of AR is important for AR N-C and AR-cofactor interactions [5], AF-2 was used as a target for computer modeling with screening for interacting candidate chemicals. The top 10 candidates are presented in Figure 2A. We had tested the effects of 8 of these 10 candidates on AR transcriptional reporter assay and found some of these candidates could really suppress AR transcriptional activity as shown in Figure 2B. Minoxidil also presented in the top 30 candidates and had been demonstrated to suppress AR related function [18].

**Figure 3:**
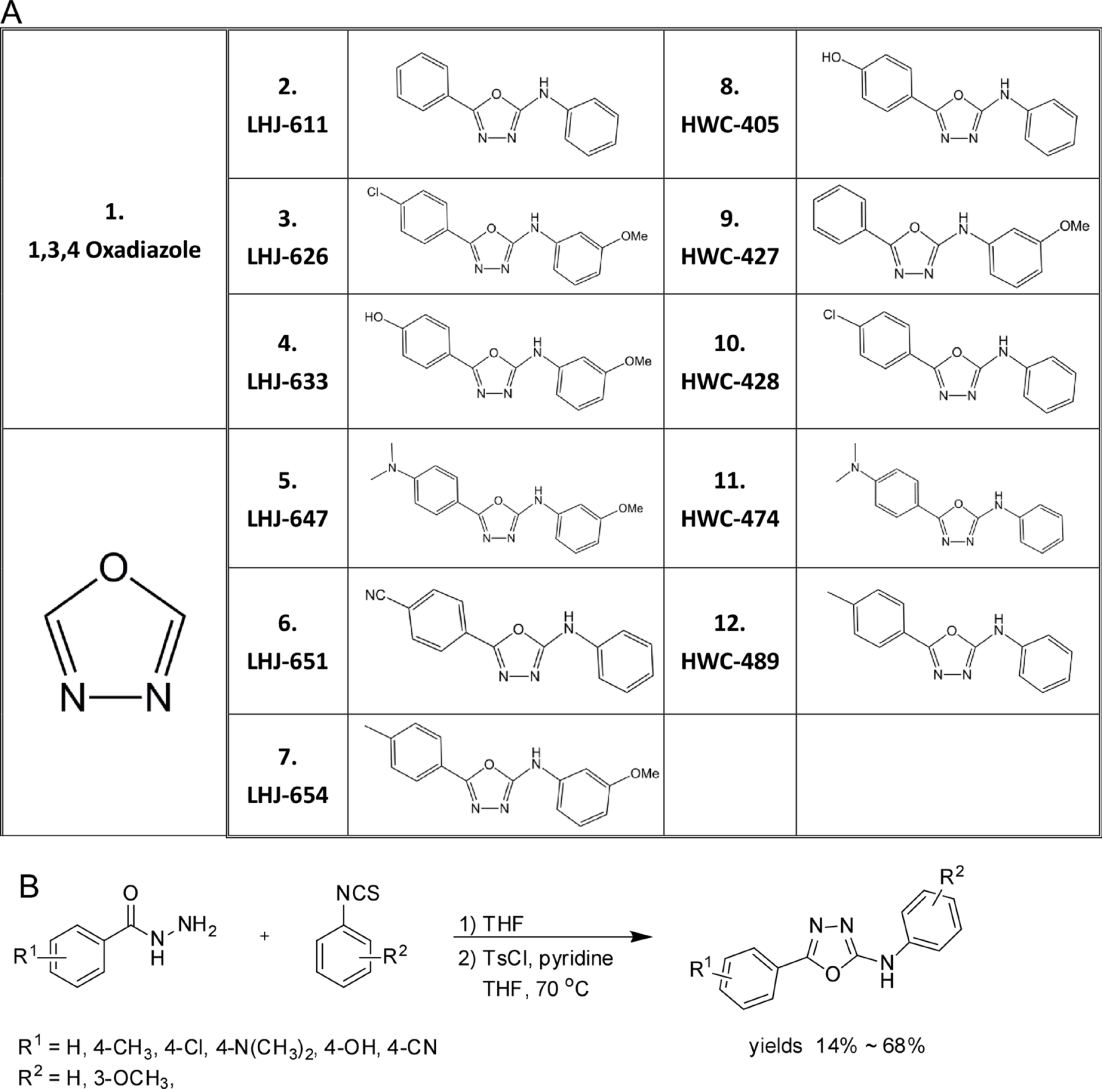
Structures and preparation of oxadiazole and derivatives (**A**) Structures of oxadiazole and derivatives (**B**) Preparation of oxadiazole derivatives. A small molecular compound library of oxadiazole derivatives was prepared as a one-pot synthesis by modification of previously reported procedures [32]. In brief, benzohydrazide was reacted with phenyl isothiocyanate to generate thiosemicarbazide, which was further converted to oxadiazole by the addition of tosyl chloride and pyridine. The yields of the desired products obtained ranged from 14% to 68%.

**Figure 4:**
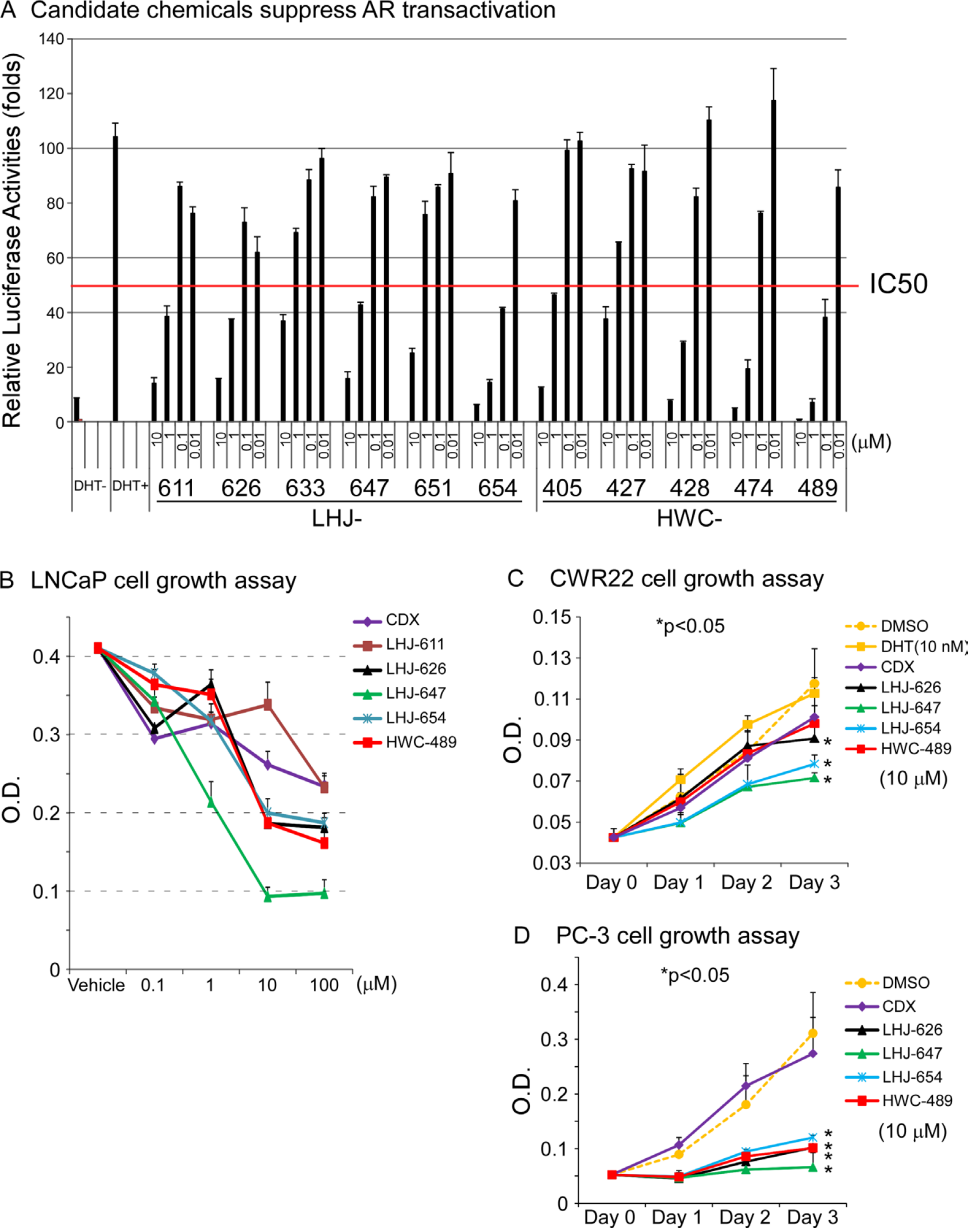
Oxadiazole derivatives suppress AR-related function *in vitro* (**A**) The candidate chemicals suppressed AR transactivation. The PC-3 prostate cancer cell line was used for experiments. The procedure was similar to that for Figure 1C. Cells were treated with ethanol, DHT or different concentrations of chemicals for 16 h. The candidate chemicals suppressed prostate cancer cell growth *in vitro*, using CDX as a control, LNCaP (**B**) CWR22R (**C**) and PC-3 (**D**).

Oxadiazole was selected as a core linker in combination with two hydrophobic rings to mimic the common core structure of these candidates and AR AF-2 interacting peptides, and a small derivative library subsequently generated, as shown in Figure 3A. The pathway of synthesis of oxadiazole derivatives is presented in Figure 3B, and results validating successful synthesis included in Supplementary Figure 1. Initially, we tested the effects of these chemicals on AR transactivation in PC-3 cells using the AR transcriptional reporter assay. The majority of test candidates bound AR with IC_50_ values of ∼1 µM, as shown in Figure 4A. The stronger candidates were further examined in the *in vitro* prostate cancer cell line growth assay. LHJ-647 and HWC-489 exerted the most potent suppressive effects with IC_50_ values of ∼1–10 µM (Figure 4B). Experiments were further performed using CWR22R (with endogenous AR) and PC-3 (without endogenous AR). The majority of candidate chemicals suppressed growth of both cell lines at a concentration of 10 µM, as shown in Figure 4C and 4D. Notably, the inhibitory effects of the candidate chemicals on prostate cancer cell line growth did not appear AR-specific.

**Figure 5:**
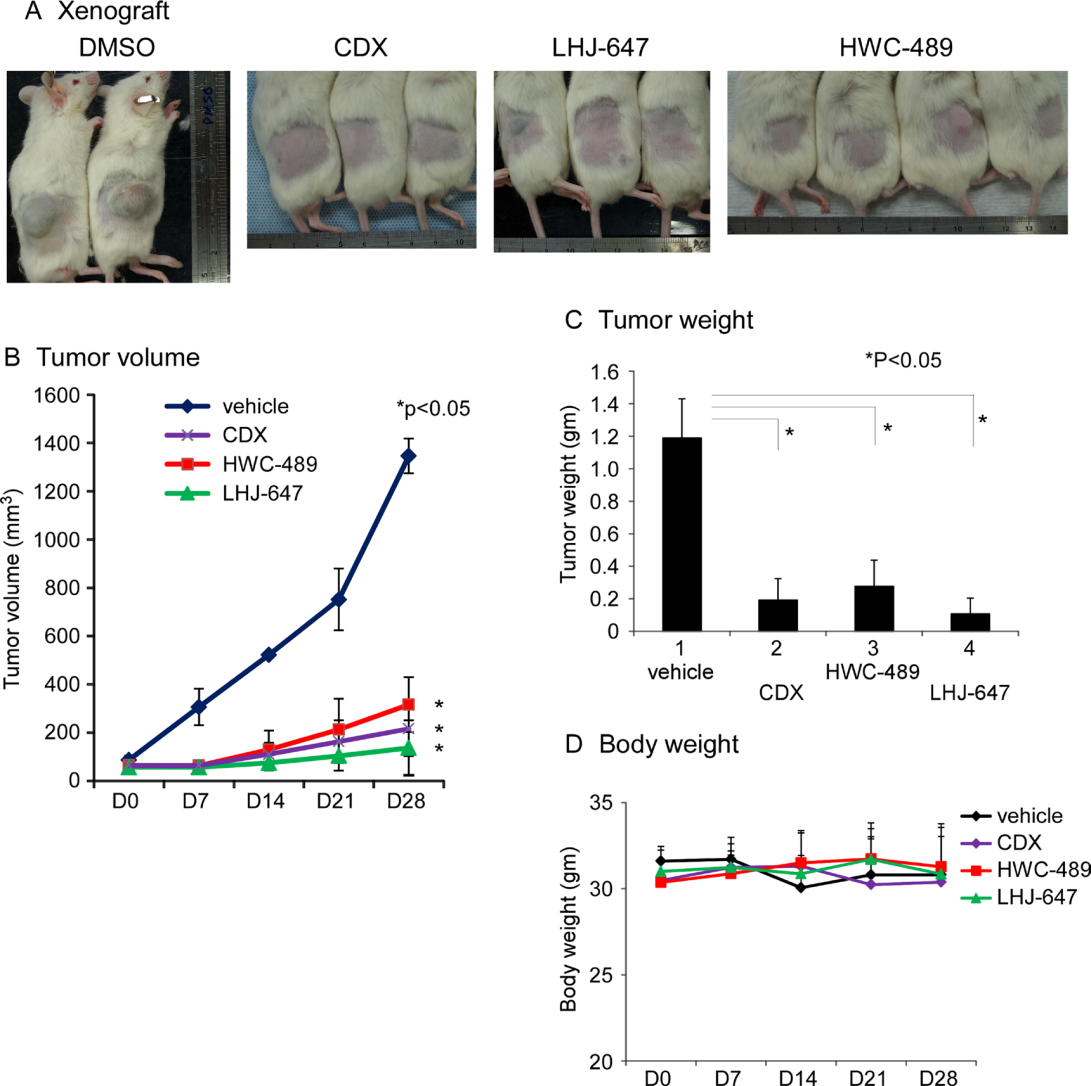
Oxadiazole derivatives suppress AR-related function *in vivo* (**A**) The mice with LNCaP cell xenograft after 28 days of treatment with DMSO, CDX, LHJ-647, and HWC-489. (**B**) Tumor volumes measured during treatment in NOD/SCID mice. HWC-489, LHJ-647, and CDX exerted significant effects, compared to vehicle treatment. (**C**) Tumor weights measured after sacrificing mice on day 28 of treatment. (**D**) Mouse body weights of all four groups displayed no significant changes during the treatment period.

These two candidates, LHJ-647 and HWC-489, additionally suppressed prostate cancer cell xenografts harboring endogenous AR and growth in a NOD/SCID mouse model with statistical significance, based on comparisons of tumor volume (Figure 5B), tumor weight (Figure 5C). No mouse mortality was observed during the experiment. Monitoring of changes in body weight, as shown in revised Figure 5D, clinical signs and gross morphology revealed no significant alterations during the study period. In the cell line study, candidate chemicals were added directly into culture medium, potentially leading to better drug penetration. However in the animal study, candidate chemicals were injected into the peritoneal cavity and circulated around the whole body. During this process, the drug may be metabolized by liver or other organs. Furthermore, individual chemicals have different pharmacokinetic profiles. These differential factors may, at least in part, contribute to the conflicting results obtained with cell line and animal studies.

**Figure 6:**
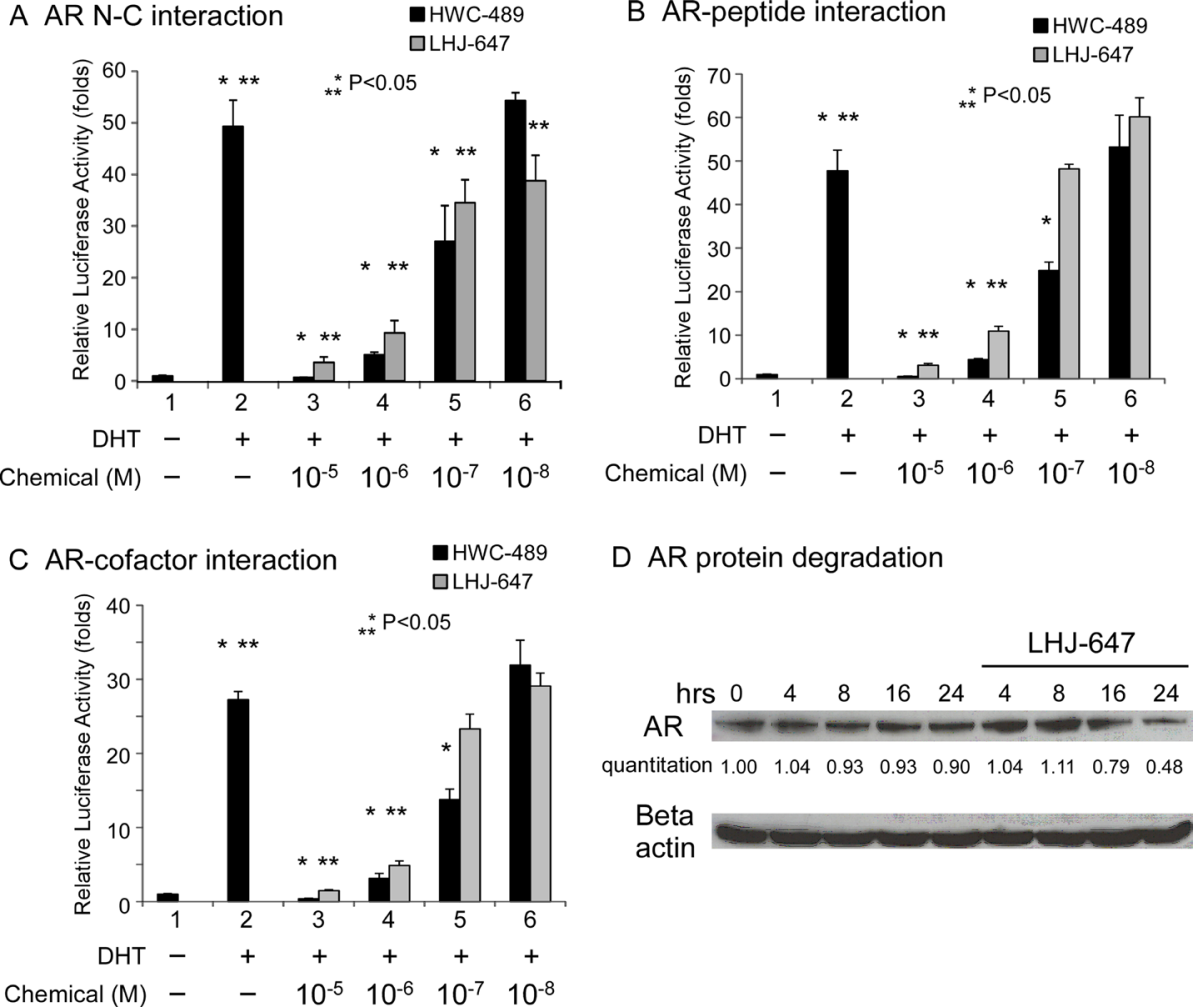
Mechanism underlying suppression of AR-related functions by oxadiazole derivatives (**A–C**) Transcriptional activity in reporter assays. PC-3 cells in 24-well plates were transfected as indicated below. After incubation for 16 h, cells were treated with ethanol, 10 nM DHT, or 0.01–10 μM HWC-489 or LHJ-647 for an additional 16 h. Luciferase activity in cell lysates was determined and normalized to protein concentrations. Relative luciferase activity was calculated using the luciferase reporter assay system [18]. (A) PC-3 cells were co-transfected with 350 ng pCDNA3-flag-hAR-N (residues 1–506), 350 ng pCDNA3-hAR-C (residues 556–919), and 300 ng MMTV-Luc plasmids. (B) PC-3 cells were co-transfected with 350 ng GAL4-DBD-3-18, 350 ng pCMX-VP16-AR, and 300 ng pG5-Luc plasmids. (C) PC-3 cells were transfected with 350 ng GAL4-DBD-ARA54C, 350 ng VP16-AR, and 300 ng pG5-Luc plasmids. (**D**) LHJ-647 promotes AR protein degradation.

In terms of mechanism of action, LHJ-647 and HWC-489 were designed to suppress the AF-2-related function of AR. Accordingly, we examined the effects of the two compounds on AR N-C, AR-peptide, and AR-cofactor interactions. Both chemicals suppressed these interactions to a significant extent within a range of 0.1–1 µM (Figure 6). LHJ-647 promoted AR protein degradation, as shown in Figure 6D.

**Figure 7:**
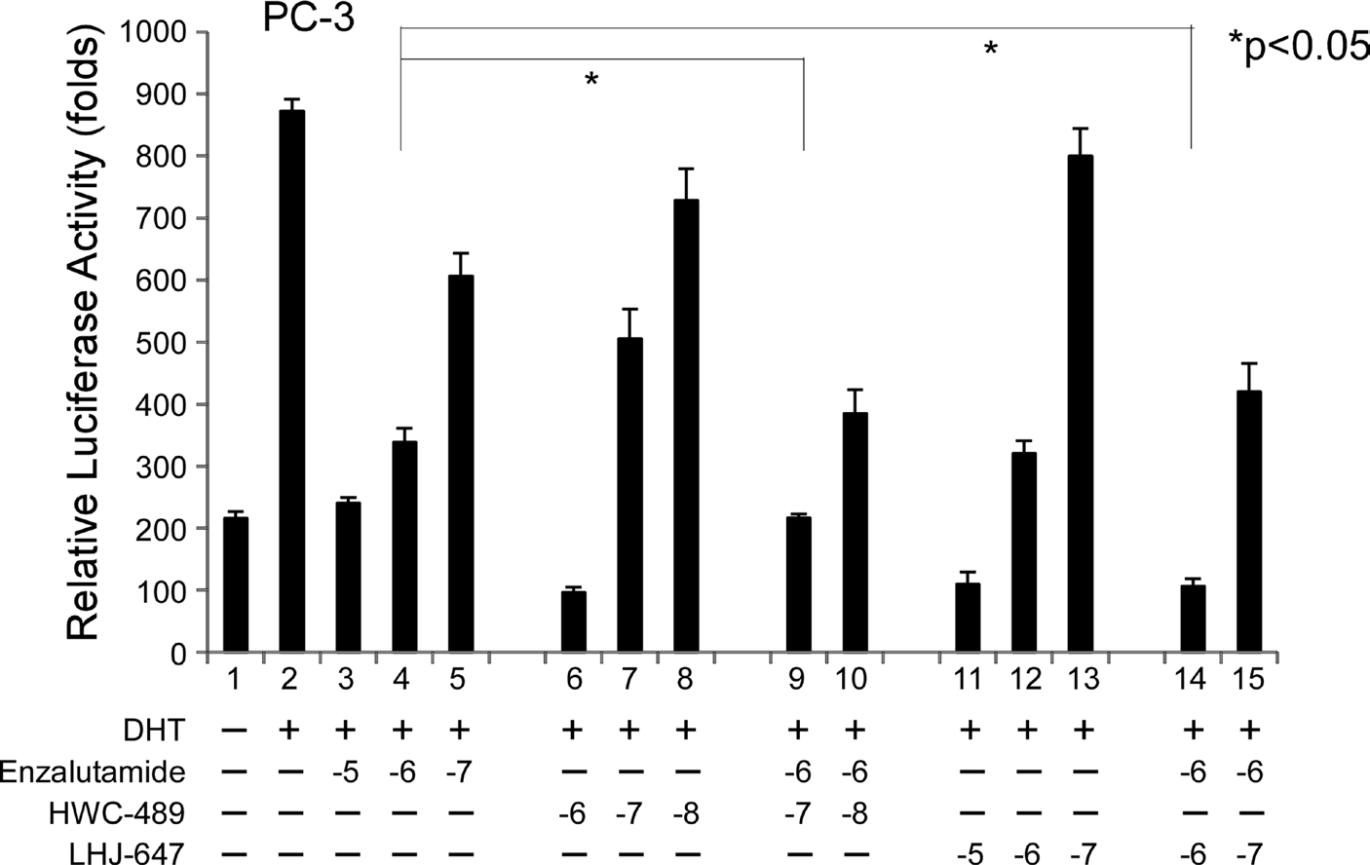
Addictive effects of oxadiazole derivatives and enzalutamide on AR transcriptional activity The PC-3 prostate cancer cell line was used for experiments. The procedure used was similar to that for Figure 1C. Cells were treated with ethanol or 10 nM DHT in the absence or presence of different concentrations of candidate chemicals and enzalutamide for 16 h.

Since LHJ-647 and HWC-489 appear to target the AF-2 site of AR distinct from traditional antiandrogens, we were interested in determining whether a combination of chemicals from these two categories can exert an additive effect on AR-related functions. In our experiments, enzalutamide suppressed AR transactivation at a concentration of 1 µM, and further addition of 1 µM LHJ-647 (lane 9 vs. 4) or 0.1 µM HWC-489 (lane 14 vs. 4) clearly induced an additive effect, as shown in Figure 7.

The collective data support a role of RIPK1 as a novel AR coregulator. Effective targeting of AF-2 of AR presents a new direction for anti-AR drug design that could be integrated in traditional prostate cancer treatment regimens.

## DISCUSSION

### RIPK1 function

RIPK1 plays crucial roles in inflammation and cell death, depending on the cell context and posttranslational modifications [17]. Upon ligand stimulation of FAS and tumor necrotic factor (TNF) receptor 1, RIPK1 is recruited to the intracellular death domain of these receptors via interactions between death domains [19]. Through RIPK1 signaling, cells undergo either survival or death pathways by interactions involving different functional domains. The adaptor function of RIPK1 is important for activation for MAPK and NF-ΚB pathways, resulting in antiapoptosis. The kinase activity of RIPK1 is involved in both apoptosis and necroptosis [20]. Homozygous knockout of RIPK1 in mice is reported to cause death shortly after birth, with disseminated inflammation and cell death in multiple organs [21]. However, mice with homozygous mutations inducing deletion of the kinase activity of RIPK1 are developmentally normal [22]. A combination of simvastatin and metformin has been shown to increase RIPK1 and RIPK3 protein expression and induce necrosis in castration-resistant prostate cancer (CRPC) cells [23]. In response to sorafenib treatment in Atg5-deficient DU145 prostate cancer cells, formation of autophagosomes could promote interactions of p62 with RIPKs, leading to cell death via necroptosis [24]. In the current study, RIPK1 interacted with AR via its FxxFY motif and suppressed AR transactivation in a dose-dependent manner. The death domain of RIPK1 was involved in its inhibitory action on AR transactivation. Expression of RIPK1 in prostate gland disease was higher in benign prostate hyperplasia and non-cancer tissue than the tumor component. Our data provide new insights into the mechanisms underlying AR functions in inflammation/cell death in prostate disease.

### AR-targeting peptidomimetics

In addition to traditional antiandrogens, a number of peptides/molecules suppress AR-mediated functions by directly targeting the AF-2 cofactor-binding pocket [16, 25, 26, 27]. Based on a pyrimidine core system, a structure-based peptidomimetic approach was used to generate AF-2 pocket-blocking chemicals exerting promising AR suppression effects [28]. Another small-molecule LxxLL mimetic, D2, displays high anti-AR efficacy at low concentrations but may potentially influence the functions of other steroid hormone receptors [29]. Combined data from phage display screening and crystal structure analyses of the AR cofactor-binding groove, FxxLF-like motif peptides provided evidence that these motifs, in particular, FxxFY, suppress AR-related functions [16]. Oxadiazole with a heterocyclic nucleus had four isomers and 1,3,4-oxadiazole derivatives exhibited a wide spectrum of biological activities, including antibiotic, anti-inflammation, herbicidal, pesticide, anticonvulsant, and anti-cancer properties [30, 31]. Oxadiazole derivatives examined in the current study disrupted AR N-C and AR-cofactor interactions and suppressed AR transcriptional activity as well as AR-mediated cancer cell growth, both *in vitro* and *in vivo*. Furthermore, this group of chemicals synergistically inhibited AR-related functions in combination with antiandrogens. Our collective findings provide new insights for the design and development of highly specific and efficient AR-targeting small molecules.

## MATERIALS AND METHODS

### Materials and plasmids

5α-Dihydrotestosterone (DHT), bicalutamide (CDX), and enzalutamide were obtained from Sigma Chemical Co. (St. Louis, MO). The Ph.D.-12 peptide library was purchased from New England Biolabs (Beverly, MA). Human cell lines (PC-3, LNCaP, CWR22R, MCF-7 and 293T) were purchased from the American Type Culture Collection (Manassas, VA). AR expression plasmids, PCMV-Flag-AR and pCMX-VP16-AR, were constructed as described previously [9, 33]. RIPK1 cDNA, prepared from human MCF-7 cells, was cloned into the p3xFLAG-CMV vector (Sigma Chemical Co.). Motif mutants of the RIPK1 plasmid, p3xFLAG-mt-AxxAA, were generated using the site-directed mutagenesis kit from Stratagene (La Jolla, CA). Anti-AR (C19) and anti-RIPK1 antibodies were purchased from Santa Cruz Biotechnology (Santa Cruz, CA).

### Mammalian two-hybrid, transfection, and reporter gene assays

The assay procedures were performed as described previously [8]. The PC-3 cell line was used for the mammalian two-hybrid and reporter gene assays.

### Co-immunoprecipitation

Details of the co-immunoprecipitation (Co-IP) procedure are described in earlier studies [16, 34]. 293T cells were employed for our experiments. Anti-Flag, anti-AR (C19), and anti-RIPK1 antibodies were purchased from Santa Cruz Biotechnology (Santa Cruz, CA).

### Glutathione Sepharose transferase (GST) pull-down assay

The detailed procedures are described in previous reports [8, 16]

### Patient enrollment

Primary prostate tumors were collected from patients subjected to prostate tumor biopsy from 2007 to 2009. Written informed consent was obtained from all participants prior to surgery. The study was approved by the Institutional Review Board of Chang Gung Memorial Hospital, Taoyuan, Taiwan.

### Cloning, expression, and purification of human AR proteins and phage display

AR DBD-LBD cDNA (residues 548–919) and AR LBD cDNA (residues 663–919) were amplified from the AR expression vector, pSG5-AR, via polymerase chain reaction (PCR), and inserted into pET28c (Novagen, San Diego, CA). The pET28c-W741L AR DBD-LBD vector was generated using the Stratagene site-directed mutagenesis kit. Protein expression and purification and phage display procedures were performed as described previously [16, 35].

### Computer model screening of chemicals binding the AR-LBD AF-2 pocket

The high- throughput screening (HTS) ligand library was obtained from the ZINC database [36], with filtering by drug-like subset and sub-structure with benzene rings at both ends. In total, ∼3000 compounds with the MOL2 format were selected for subsequent virtual docking. The protein structure was generated with Chimera software using the AR-LBD-DHT tertiary complex (pdb code: 4EOA) as the template, followed by adding hydrogen, protonation, ionization and energy minimization with the CHARMm force field to optimize the geometry of the residues. Virtual docking was carried out with the GOLD program (ver. 2.0) using the prepared ligands. The ligand area of the AF-2 site was defined within a 10 Å radius surrounding the sulfur atom of the Met894 residue. Ligands were additionally constrained with a protein H-bond to E897 and K720 residues. Candidates were ranked using the GOLDscore function, and the top 10 compounds selected for the AR functional assay.

### Oxadiazole derivative preparation

A solution of benzohydrazide (1 eq) and phenyl isothiocyanate (1–1.2 eq) in THF was stirred overnight at room temperature. Toluenesulfonyl chloride (1.2 eq) and pyridine (2 eq) were added to the reaction mixture and heated to 70°C for 18–24 h. After stirring for 24 h, pyridine and THF were removed under reduced pressure [32]. The residue was dissolved in ethyl acetate and washed with 10% HCl _(aq)_. The organic layer was dried over MgSO_4_, and then filtered and concentrated *in vacuo*. The residue was purified using silica gel column chromatography to obtain the desired product with 14–68% yield. The synthesis pathways of these chemicals are shown in Supplementary Figure 1.

### Cell growth assay

LNCaP, CWR22R, and PC-3 cells were grown in RPMI containing 10% FBS. Cells were plated at a density of 5 × 10^4^ cells/well in 24-well plates overnight and incubated with difference concentrations of chemicals for 48 h. Cell growth was assessed using the 3-{4,5-dimethylthiazol-2-yl}-2,5-diphenyltetrazolium bromide (MTT) assay [25]. At each time-point, 50 μl of 5 mg/ml MTT was added to each well containing 500 μl medium and incubated for 3 h, followed by the addition of 500 μl isopropyl alcohol to dissolve the reduced formazan product. Absorbance of each well was measured at a wavelength of 590 nm in a DU 640B spectrophotometer (Beckman, Fullerton, CA), according to the manufacturer’s protocol. Values represent mean OD_590_ ± SD from at least three independent reaction wells.

### Animal studies

All experiments involving laboratory animals were conducted in accordance with the Guidelines for Animal Experiments of Chang Gung Memorial Hospital and approved by the Animal Research Committee at the hospital. LNCaP cells were harvested, washed twice with phosphate-buffed saline, and resuspended at a final concentration of 5 × 10^6^ cells/mL in Matrigel (BD Biosciences, San Jose, CA) containing basement membrane components. Next, 5 × 10^5^ cells (100 μL per site) were subcutaneously injected into the flanks of 4–6 week-old male NOD/SCID mice (BioLASCO, Taiwan). Tumor development was confirmed within 2–3 weeks after injection of the same number of cell subclones. Treatment with different chemicals was initiated at the same time. Tumor dimensions were measured twice a week with calipers, and tumor volumes calculated using the formula: tumor volume (mm^3^) = tumor length (mm) × [tumor width (mm)]^2^ × 0.5. Three to five mice were used per group (with or without chemical treatment). Mice were sacrificed 4 weeks after chemical injection [37] and tumors harvested for further analyses. For evaluation of treatment toxicity, animal mortality, changes in body weight, clinical signs and gross morphology were monitored during the experiment.

### Statistical analysis

Data are presented as means ± SEM. Differences between two groups were assessed using the unpaired two-tailed Student’s *t* test.

## SUPPLEMENTARY MATERIALS FIGURE


