# Supplementary material for: Characterization of a novel androgen receptor (AR) coregulator RIPK1 and related chemicals that suppress AR-mediated prostate cancer growth via peptide and chemical screening
Source: Oncotarget. 2017 May 13;8(41):69508–19. doi: 10.18632/oncotarget.17843 (PMC5642495; doi:10.18632/oncotarget.17843)
Supplement: Supplementary file 1 [file oncotarget-08-69508-s001.pdf]

Characterization of a novel androgen receptor (AR) coregulator RIPK1 and related chemicals that suppress AR-mediated prostate cancer growth via peptide and chemical screening

Supplementary Materials

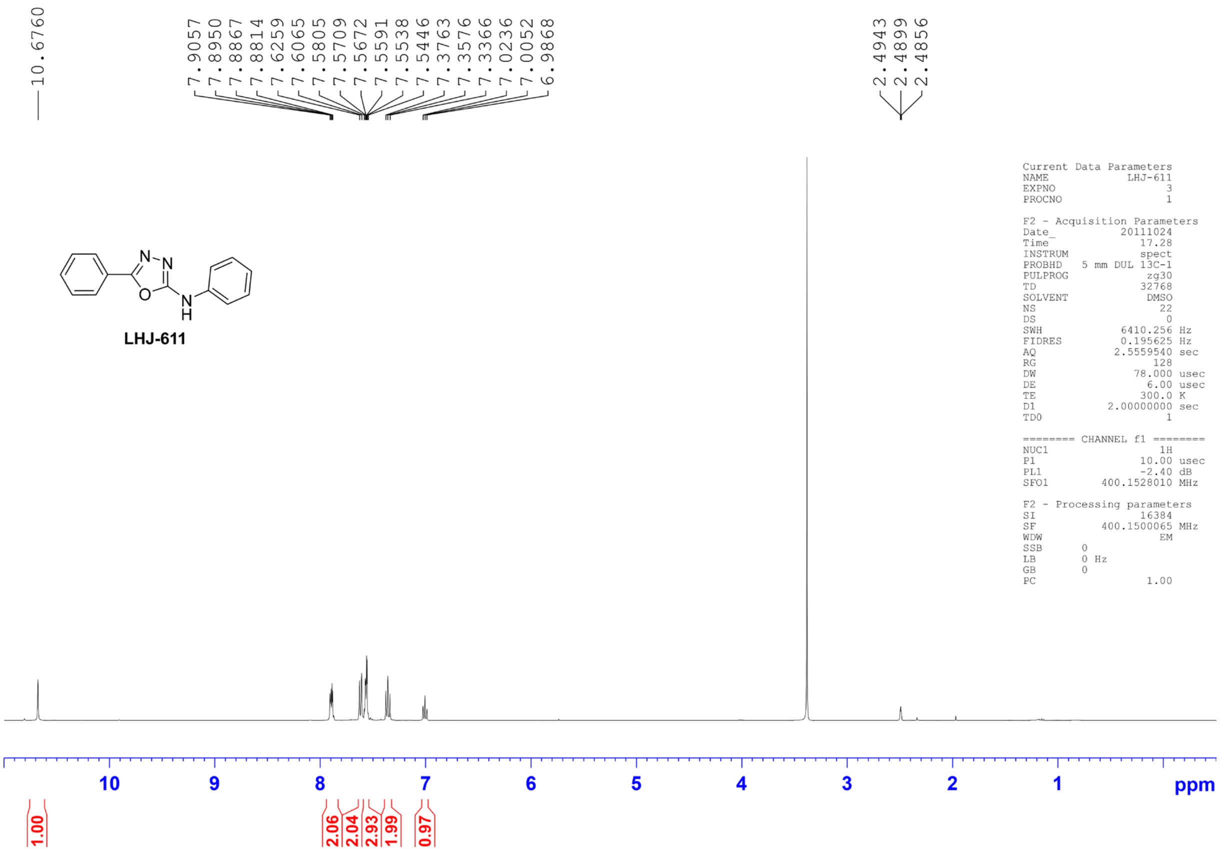

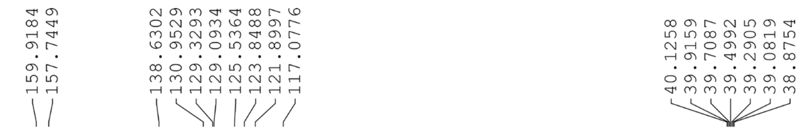

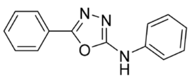

LHJ-611

```
Current Data Parameters
NAME      LHJ-611-13C.fid
EXPNO                   1
PROCNO                  1

F2 - Processing parameters
SI                  65536
SF         100.5218769 MHz
WDW                    EM
SSB      0
LB                0.30 Hz
GB       0
PC                   1.00
```

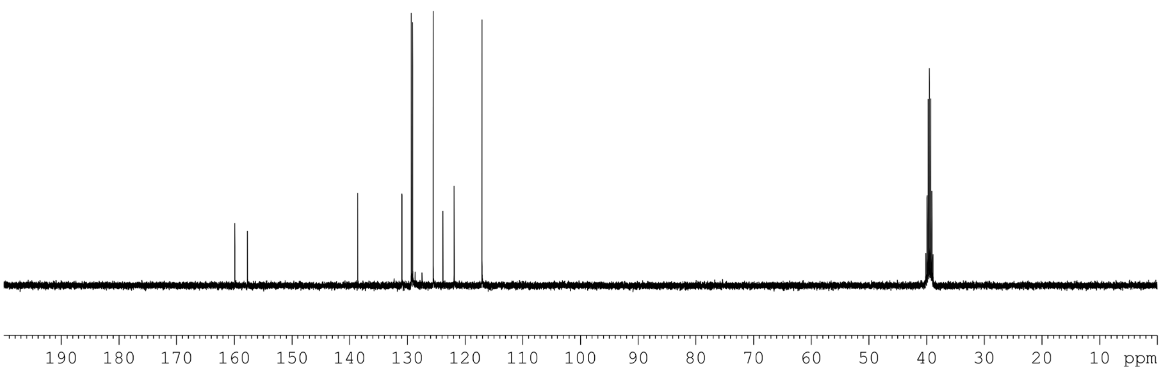

LHJ-611 (HR-ESI neg)

Elemental Composition Report

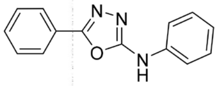

LHJ-611

Page 1

Single Mass Analysis
Tolerance = 100.0 PPM / DBE: min = -100.0, max = 1000.0
Element prediction: Off
Number of isotope peaks used for i-FIT = 2

Monoisotopic Mass, Even Electron Ions
15 formula(e) evaluated with 1 results within limits (all results (up to 1000) for each mass)
Elements Used:
C: 0-1000 H: 0-4000 N: 3-3 O: 1-1
LHJ-611

KE267

31-Oct-2011
13:46:34
2: TOF MS ES-
1.66e+004

1031_LHJ-611 34 (3.365) Cm (34:35-2)

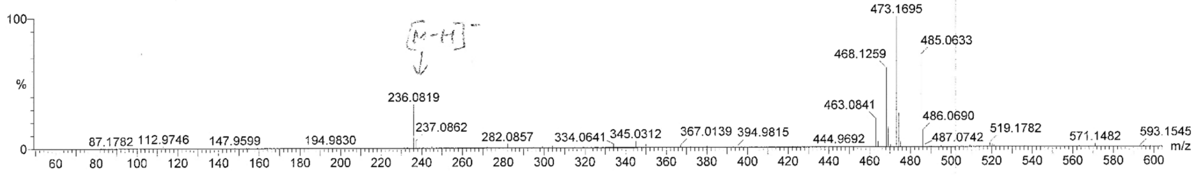

Minimum:				-100.0				
Maximum:		5.0	100.0	1000.0				
Mass	Calc. Mass	mDa	PPM	DBE	i-FIT	i-FIT (Norm)	Formula	
236.0819	236.0824	-0.5	-2.1	11.5	34.9	0.0	C14 H10 N3 O	

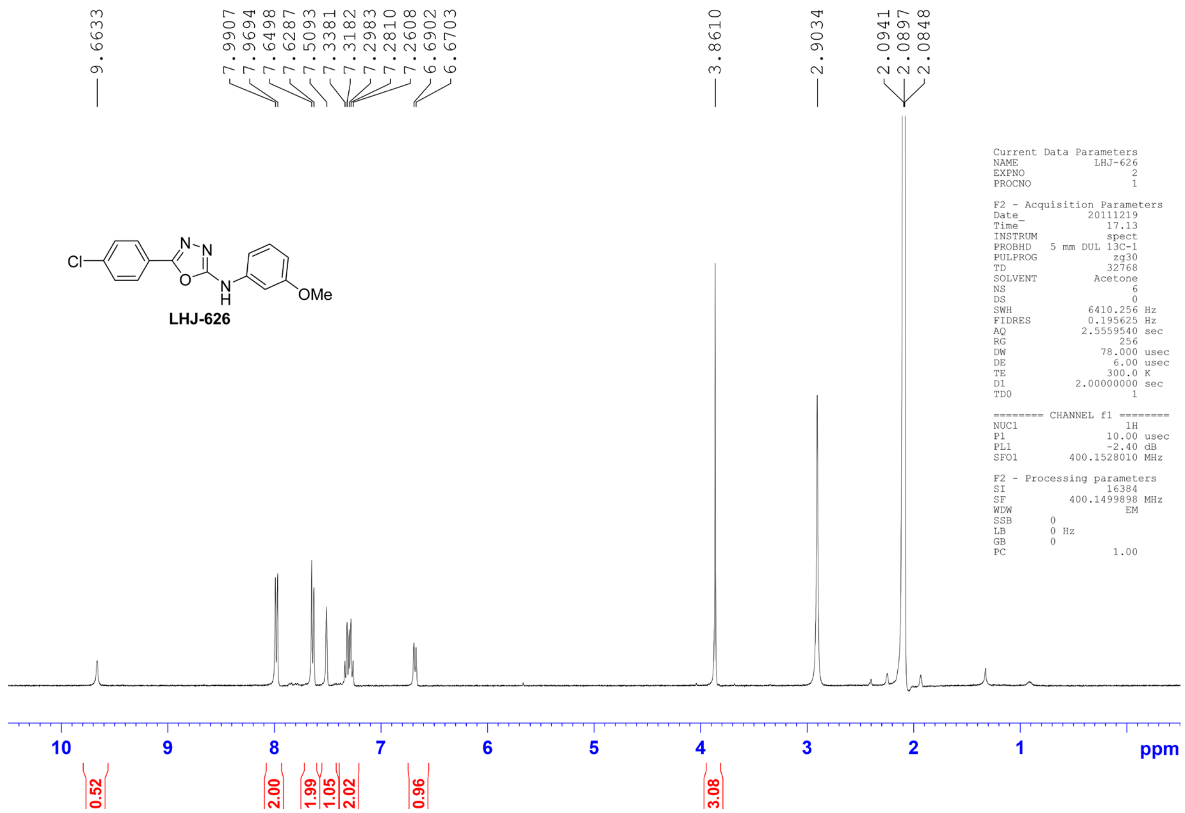
9.6633
7.9907
7.9694
7.6498
7.6287
7.5093
7.3381
7.3182
7.2983
7.2810
7.2608
6.6902
6.6703
3.8610
2.9034
2.0941
2.0897
2.0848
Cl
N
N
O
N
H
OMe
LHJ-626
Current Data Parameters
NAME LHJ-626
EXPNO 2
PROCNO 1
F2 - Acquisition Parameters
Date_ 20111219
Time 17.13
INSTRUM spect
PROBHD 5 mm DUL 13C-1
PULPROG zg30
TD 32768
SOLVENT Acetone
NS 6
DS 0
SWH 6410.256 Hz
FIDRES 0.195625 Hz
AQ 2.5559540 sec
RG 256
DW 78.000 usec
DE 6.00 usec
TE 300.0 K
D1 2.00000000 sec
TD0 1
======== CHANNEL f1 ========
NUC1 1H
P1 10.00 usec
PL1 -2.40 dB
SFO1 400.1528010 MHz
F2 - Processing parameters
SI 16384
SF 400.1499898 MHz
WDW EM
SSB 0
LB 0 Hz
GB 0
PC 1.00
10
9
8
7
6
5
4
3
2
1
ppm
0.52
2.00
1.99
1.05
2.02
0.96
3.08

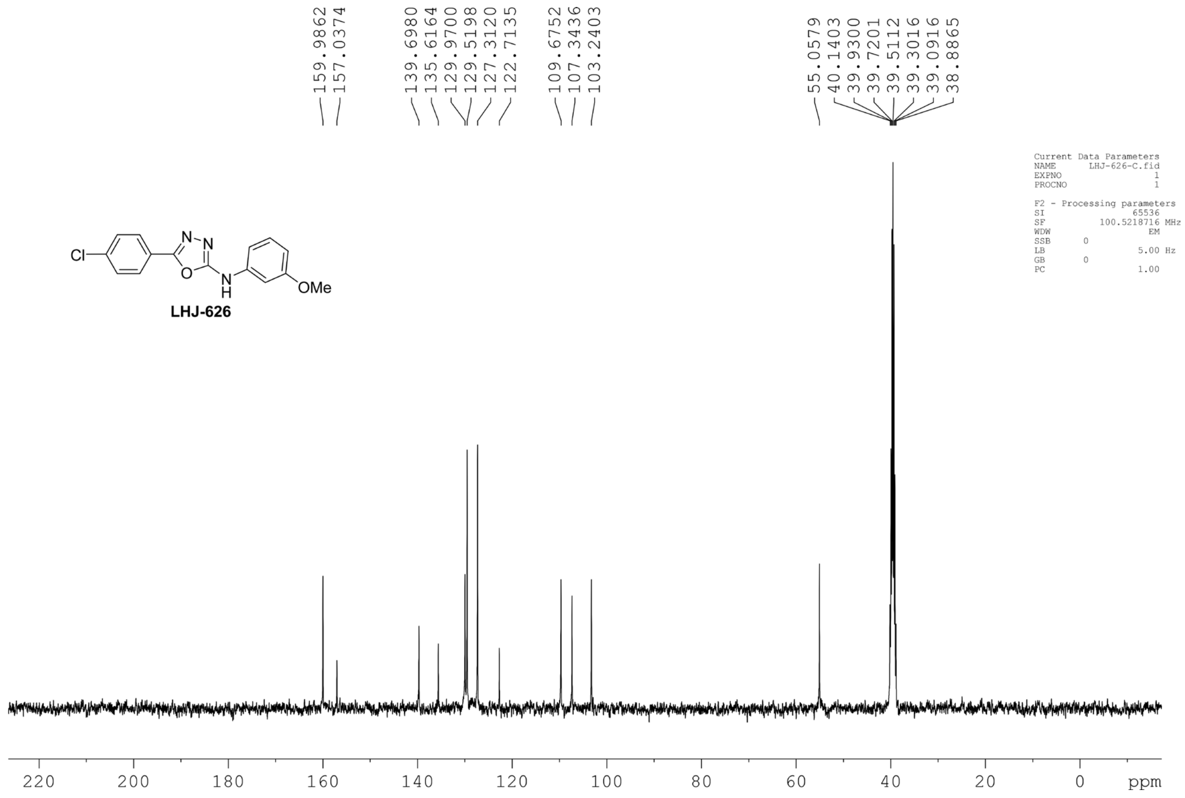
159.9862
157.0374
139.6980
135.6164
129.9700
129.5198
127.3120
122.7135
109.6752
107.3436
103.2403
55.0579
40.1403
39.9300
39.7201
39.5112
39.3016
39.0916
38.8865
Cl
N
N
O
N
H
OMe
LHJ-626
Current Data Parameters
NAME LHJ-626-C.fid
EXPNO 1
PROCNO 1
F2 - Processing parameters
SI 65536
SF 100.5218716 MHz
WDW EM
SSB 0
LB 5.00 Hz
GB 0
PC 1.00
220
200
180
160
140
120
100
80
60
40
20
0
ppm

LHJ-626 (HR-ESI neg)

Elemental Composition Report

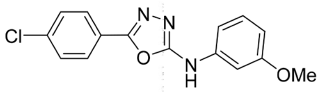

LHJ-626

Page 1

Single Mass Analysis
Tolerance = 100.0 PPM / DBE: min = -100.0, max = 1000.0
Element prediction: Off
Number of isotope peaks used for i-FIT = 2

Monoisotopic Mass, Even Electron Ions
17 formula(e) evaluated with 1 results within limits (all results (up to 1000) for each mass)
Elements Used:
C: 0-4000 H: 0-4000 N: 3-3 O: 2-2 Cl: 1-1

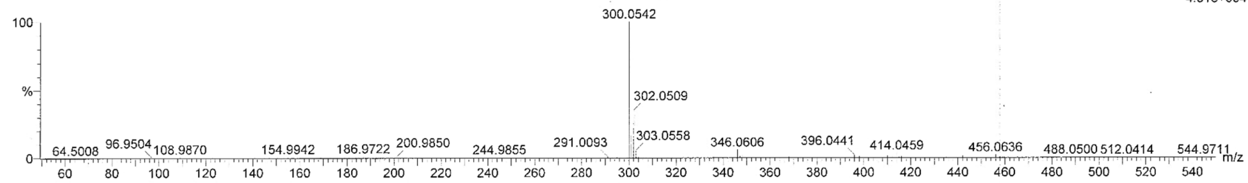

Minimum:				-100.0		
Maximum:		5.0	100.0	1000.0		

Mass	Calc. Mass	mDa	PPM	DBE	i-FIT	i-FIT (Norm)	Formula
300.0542	300.0540	0.2	0.7	11.5	102.0	0.0	C15 H11 N3 O2 Cl

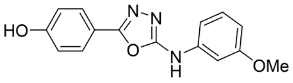

LHJ-633

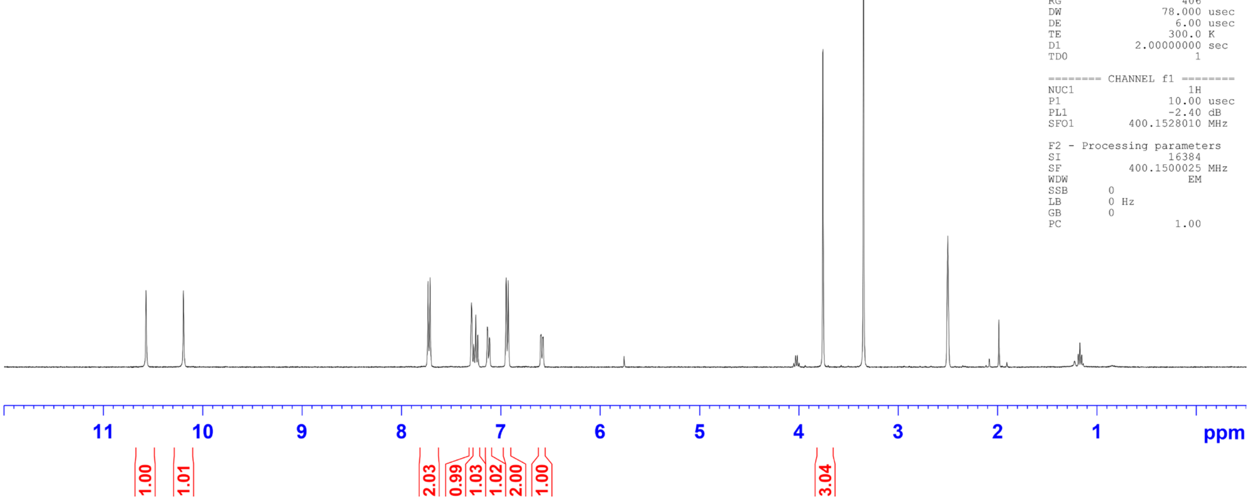

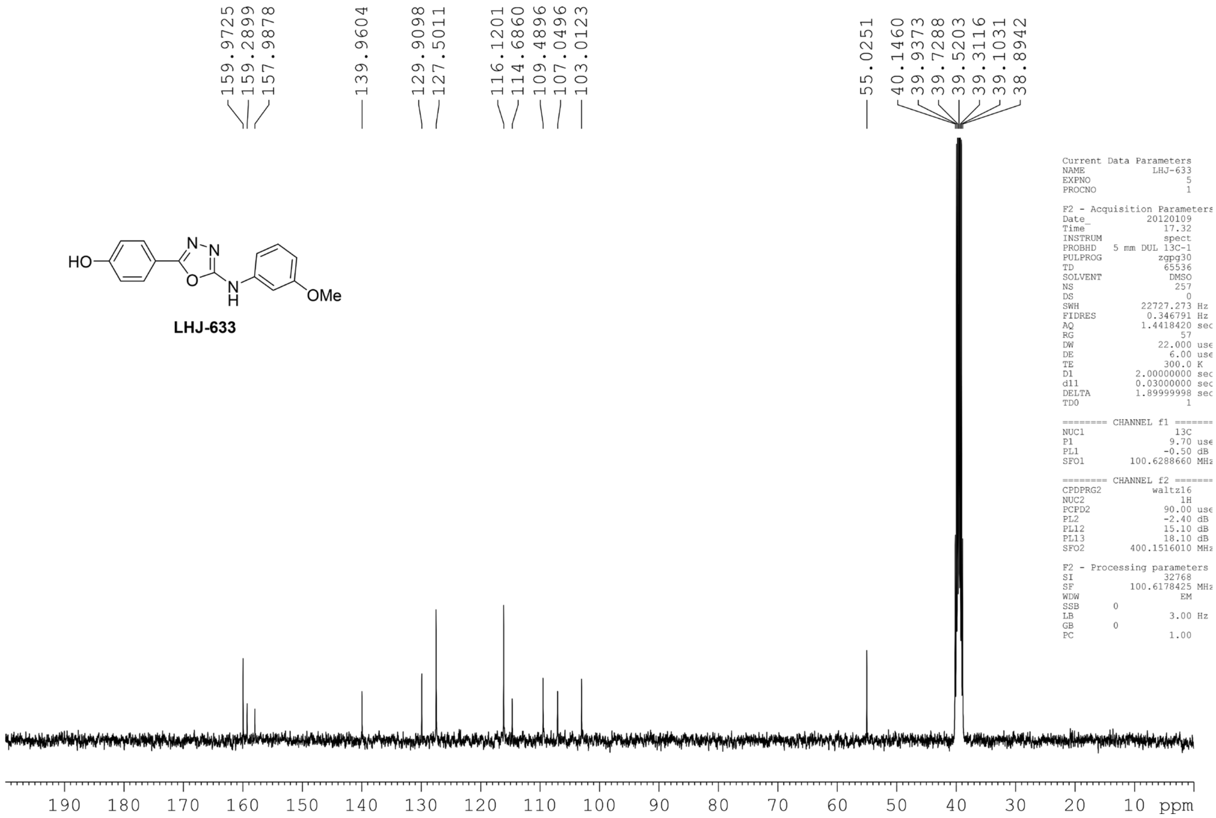

LHJ-633 (HR-ESI neg)

Elemental Composition Report

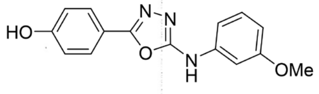

LHJ-633

Page 1

Single Mass Analysis
Tolerance = 100.0 PPM / DBE: min = -100.0, max = 1000.0
Element prediction: Off
Number of isotope peaks used for i-FIT = 2

Monoisotopic Mass, Even Electron Ions
35 formula(e) evaluated with 1 results within limits (all results (up to 1000) for each mass)
Elements Used:
C: 0-4000 H: 0-4000 N: 3-3 O: 2-3
LHJ-633

KE267

13-Jan-2012
12:56:28
2: TOF MS ES-
1.01e+004

0113_LHJ-633 21 (2.110) Cm (21-101x5.000)

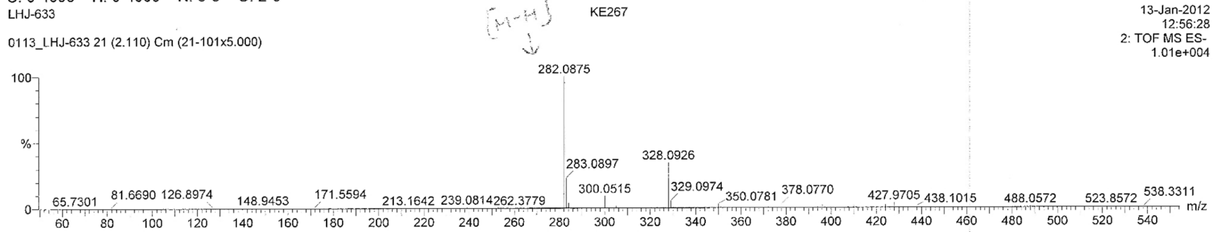

Minimum:				-100.0			
Maximum:		5.0	100.0	1000.0			
Mass	Calc. Mass	mDa	PPM	DBE	i-FIT	i-FIT (Norm)	Formula
282.0875	282.0879	-0.4	-1.4	11.5	90.1	0.0	C15 H12 N3 O3

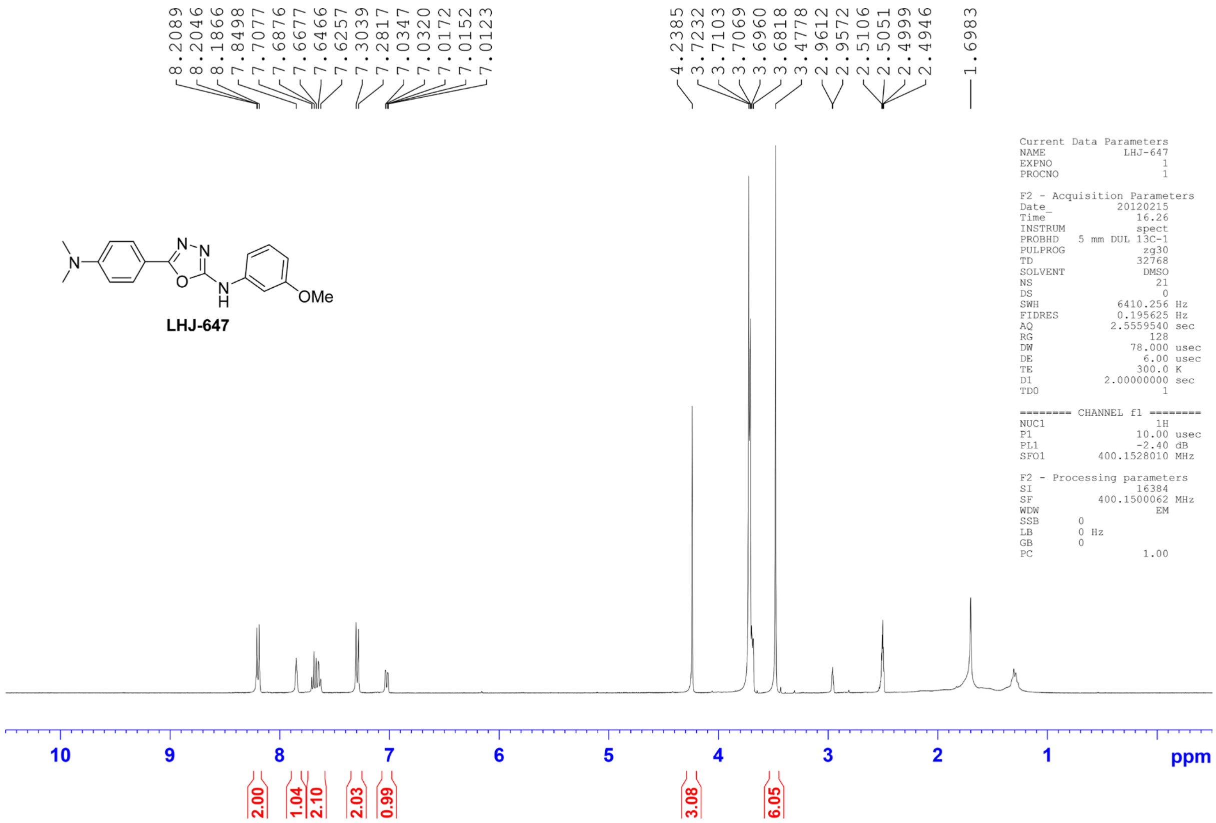
8.2089
8.2046
8.1866
7.8498
7.7077
7.6876
7.6677
7.6466
7.6257
7.3039
7.2817
7.0347
7.0320
7.0172
7.0152
7.0123
4.2385
3.7232
3.7103
3.7069
3.6960
3.6818
3.4778
2.9612
2.9572
2.5106
2.5051
2.4999
2.4946
1.6983
LHJ-647
OMe
Current Data Parameters
NAME LHJ-647
EXPNO 1
PROCNO 1
F2 - Acquisition Parameters
Date_ 20120215
Time 16.26
INSTRUM spect
PROBHD 5 mm DUL 13C-1
PULPROG zg30
TD 32768
SOLVENT DMSO
NS 21
DS 0
SWH 6410.256 Hz
FIDRES 0.195625 Hz
AQ 2.5559540 sec
RG 128
DW 78.000 usec
DE 6.00 usec
TE 300.0 K
D1 2.00000000 sec
TD0 1
======== CHANNEL f1 ========
NUC1 1H
P1 10.00 usec
PL1 -2.40 dB
SFO1 400.1528010 MHz
F2 - Processing parameters
SI 16384
SF 400.1500062 MHz
WDW EM
SSB 0
LB 0 Hz
GB 0
PC 1.00
10 9 8 7 6 5 4 3 2 1 ppm
2.00
1.04
2.10
2.03
0.99
3.08
6.05

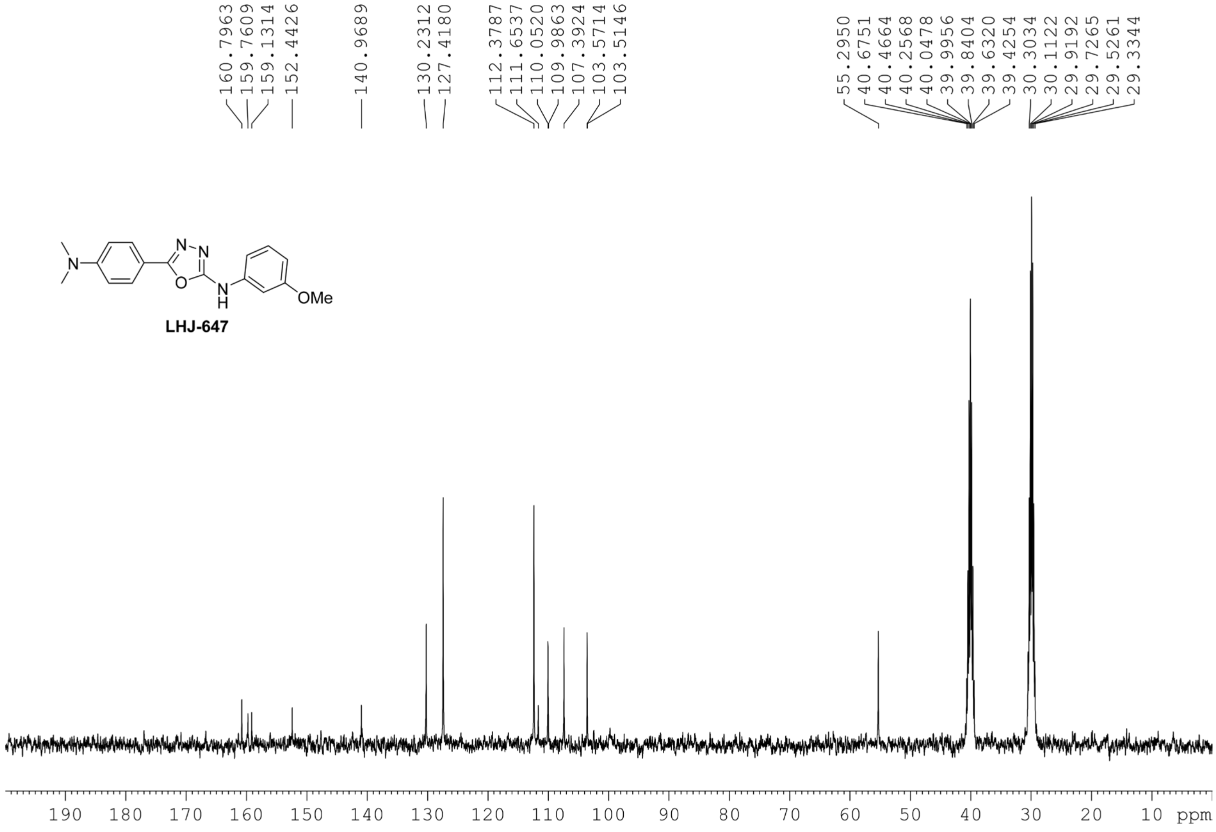
160.7963
159.7609
159.1314
152.4426
140.9689
130.2312
127.4180
112.3787
111.6537
110.0520
109.9863
107.3924
103.5714
103.5146
55.2950
40.6751
40.4664
40.2568
40.0478
39.9956
39.8404
39.6320
39.4254
30.3034
30.1122
29.9192
29.7265
29.5261
29.3344
LHJ-647
OMe
190 180 170 160 150 140 130 120 110 100 90 80 70 60 50 40 30 20 10 ppm

LHJ-647 (HR-ESIneg)

Elemental Composition Report

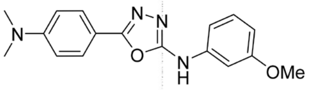

LHJ-647

Page 1

Single Mass Analysis
Tolerance = 20.0 PPM / DBE: min = -100.0, max = 1000.0
Element prediction: Off
Number of isotope peaks used for i-FIT = 2

Monoisotopic Mass, Even Electron Ions
18 formula(e) evaluated with 1 results within limits (all results (up to 1000) for each mass)
Elements Used:
C: 0-4000 H: 0-4000 N: 4-4 O: 2-2
LHJ-647

KE267

21-Feb-2012
16:14:35

0221_LHJ-647 29 (2.896) Cm (29-101x10.000)

2: TOF MS ES-
1.08e+003

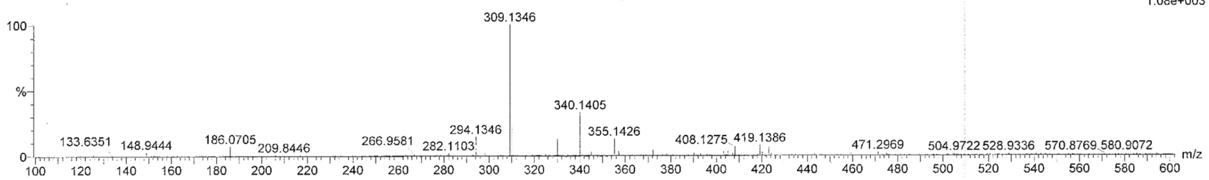

Minimum:				-100.0		
Maximum:		5.0	20.0	1000.0		
Mass	Calc. Mass	mDa	PPM	DBE	i-FIT	i-FIT (Norm) Formula
309.1346	309.1352	-0.6	-1.9	11.5	37.6	0.0 C17 H17 N4 O2

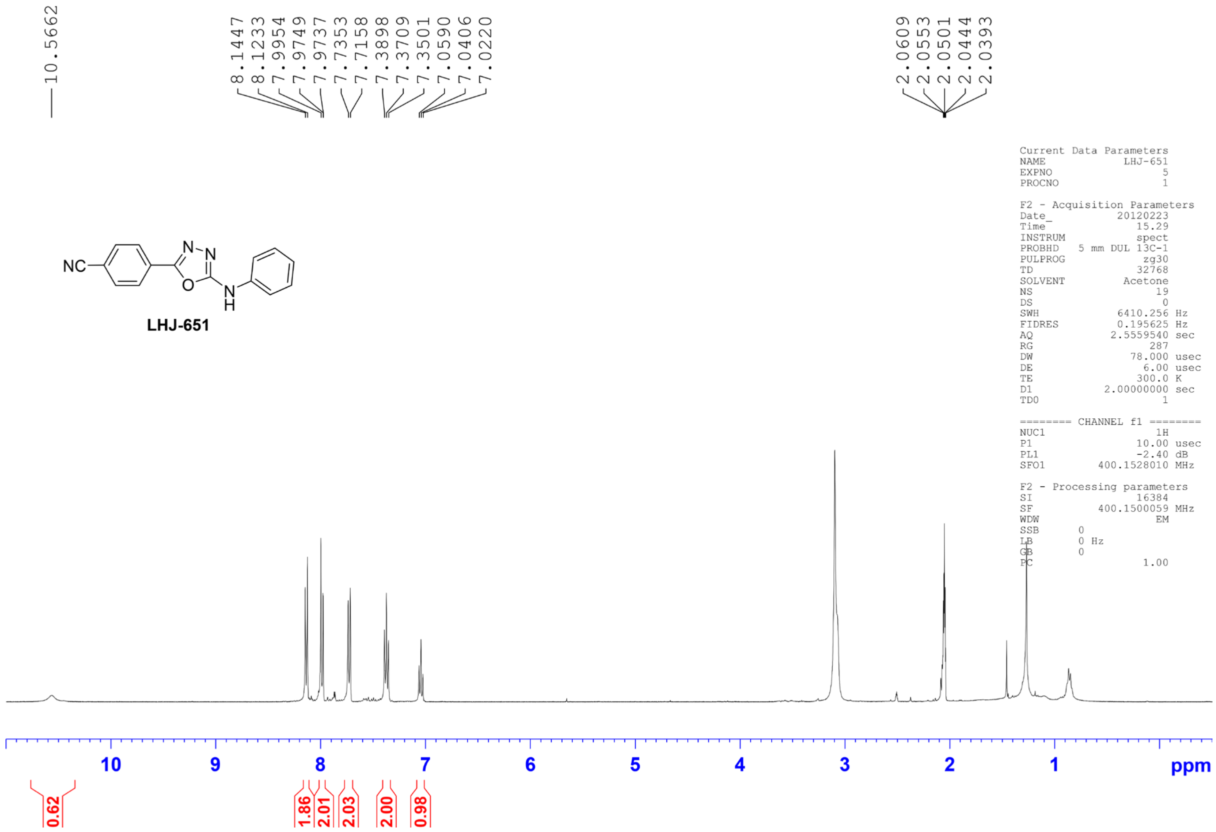

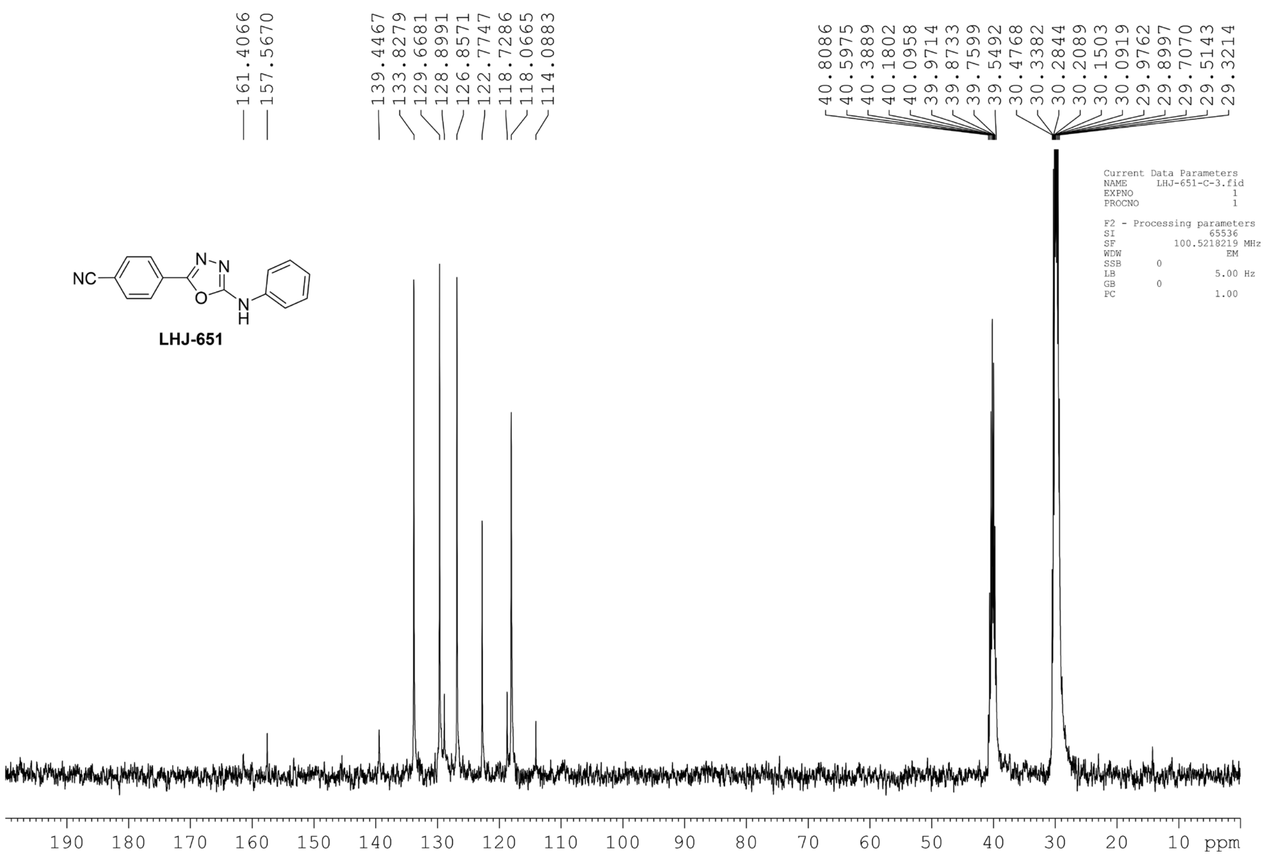

LHJ- 651 (HR-ESI neg)

Elemental Composition Report

Page 1

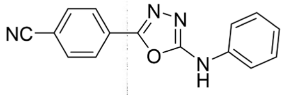

LHJ-651

Single Mass Analysis
Tolerance = 50.0 PPM / DBE: min = -100.0, max = 1000.0
Element prediction: Off
Number of isotope peaks used for i-FIT = 2

Monoisotopic Mass, Even Electron Ions
16 formula(e) evaluated with 1 results within limits (all results (up to 1000) for each mass)
Elements Used:
C: 0-4000 H: 0-4000 N: 4-4 O: 1-1
LHJ-651

KE267

28-Mar-2012
13:34:38
2: TOF MS ES-
3.87e+004

0328_LHJ-651 10 (1.013)

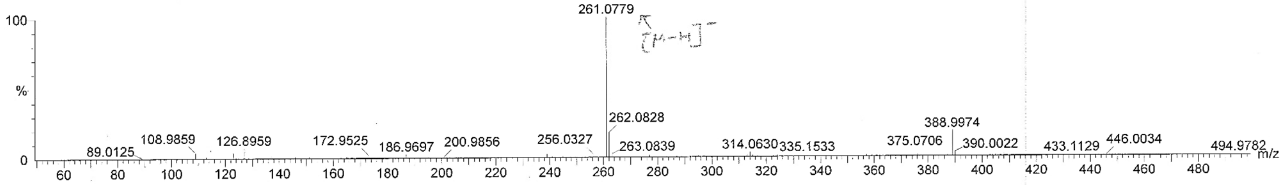

Minimum:				-100.0			
Maximum:		5.0	50.0	1000.0			
Mass	Calc. Mass	mDa	PPM	DBE	i-FIT	i-FIT (Norm)	Formula
261.0779	261.0776	0.3	1.1	13.5	81.4	0.0	C15 H9 N4 O

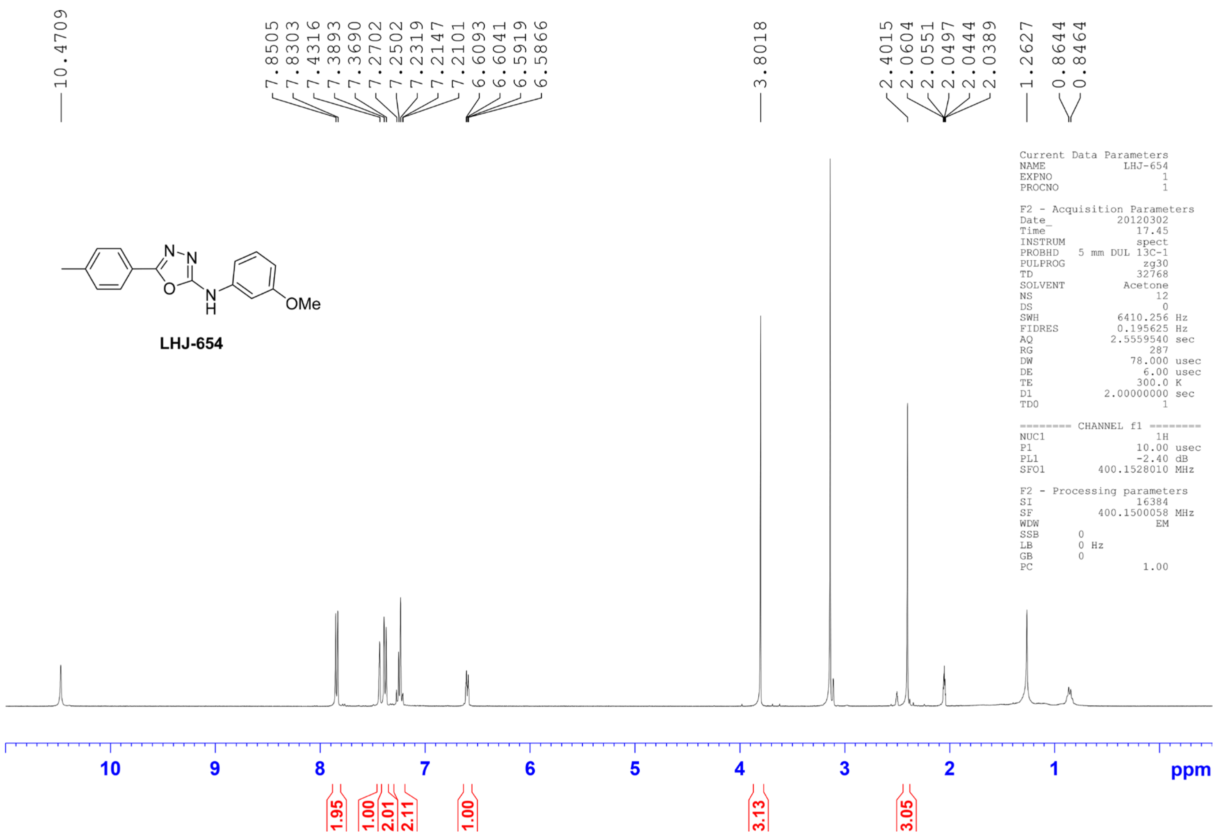
10.4709
7.8505
7.8303
7.4316
7.3893
7.3690
7.2702
7.2502
7.2319
7.2147
7.2101
6.6093
6.6041
6.5919
6.5866
3.8018
2.4015
2.0604
2.0551
2.0497
2.0444
2.0389
1.2627
0.8644
0.8464
OMe
LHJ-654
Current Data Parameters
NAME LHJ-654
EXPNO 1
PROCNO 1
F2 - Acquisition Parameters
Date_ 20120302
Time 17.45
INSTRUM spect
PROBHD 5 mm DUL 13C-1
PULPROG zg30
TD 32768
SOLVENT Acetone
NS 12
DS 0
SWH 6410.256 Hz
FIDRES 0.195625 Hz
AQ 2.5559540 sec
RG 287
DW 78.000 usec
DE 6.00 usec
TE 300.0 K
D1 2.00000000 sec
TD0 1
======== CHANNEL f1 ========
NUC1 1H
P1 10.00 usec
PL1 -2.40 dB
SFO1 400.1528010 MHz
F2 - Processing parameters
SI 16384
SF 400.1500058 MHz
WDW EM
SSB 0
LB 0 Hz
GB 0
PC 1.00
10
9
8
7
6
5
4
3
2
1
ppm
1.95
1.00
2.01
2.11
1.00
3.13
3.05

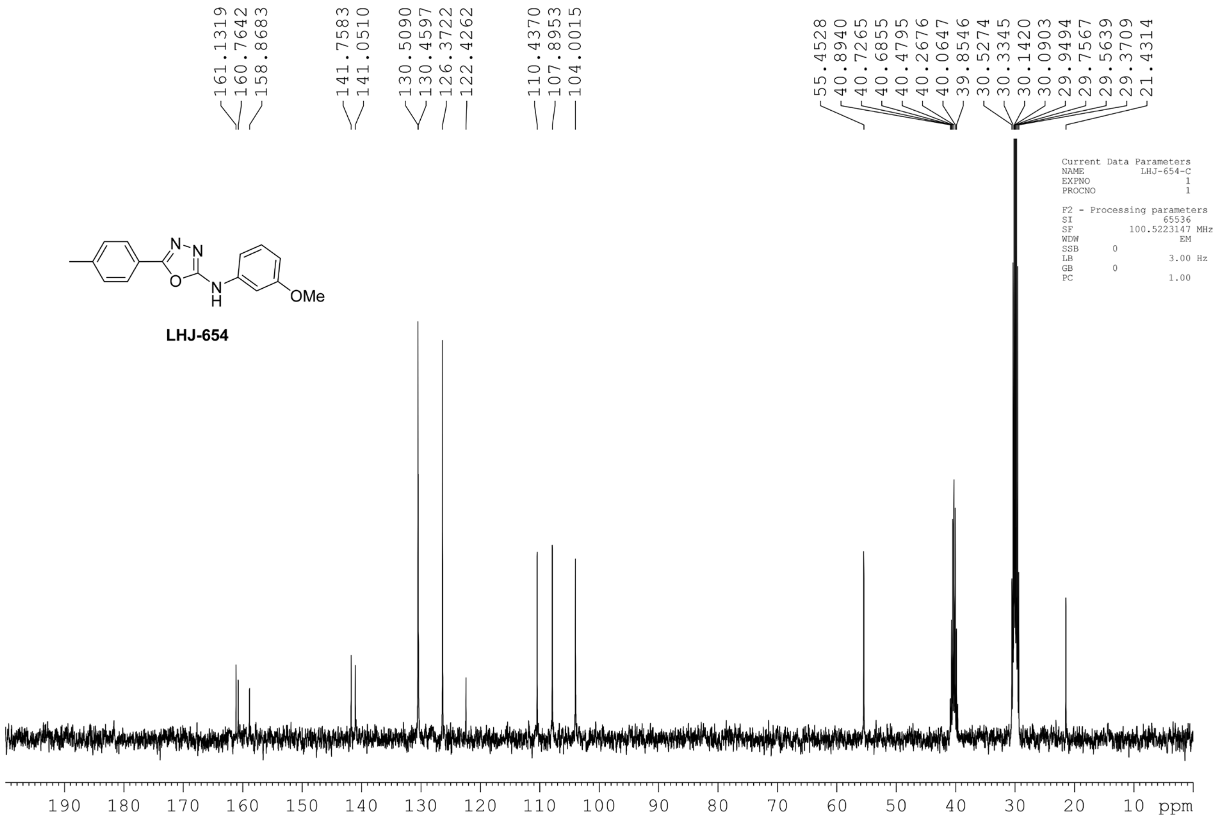
161.1319
160.7642
158.8683
141.7583
141.0510
130.5090
130.4597
126.3722
122.4262
110.4370
107.8953
104.0015
55.4528
40.8940
40.7265
40.6855
40.4795
40.2676
40.0647
39.8546
30.5274
30.3345
30.1420
30.0903
29.9494
29.7567
29.5639
29.3709
21.4314
OMe
LHJ-654
Current Data Parameters
NAME LHJ-654-C
EXPNO 1
PROCNO 1
F2 - Processing parameters
SI 65536
SF 100.5223147 MHz
WDW EM
SSB 0
LB 3.00 Hz
GB 0
PC 1.00
190
180
170
160
150
140
130
120
110
100
90
80
70
60
50
40
30
20
10
ppm

LHJ-654 (HR-ESI neg)

Elemental Composition Report

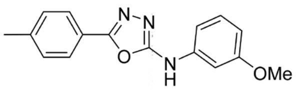

Page 1

LHJ-654

Single Mass Analysis

Tolerance = 20.0 PPM / DBE: min = -100.0, max = 1000.0
Element prediction: Off
Number of isotope peaks used for i-FIT = 2

Monoisotopic Mass, Even Electron Ions
18 formula(e) evaluated with 1 results within limits (all results (up to 1000) for each mass)
Elements Used:
C: 0-4000 H: 0-4000 N: 3-3 O: 2-2

LHJ-654 KE267 19-Mar-2012 16:03:00
0316_LHJ-654_3 2 (0.109) Cm (2-1) 1: TOF MS ES- 6.33e+003

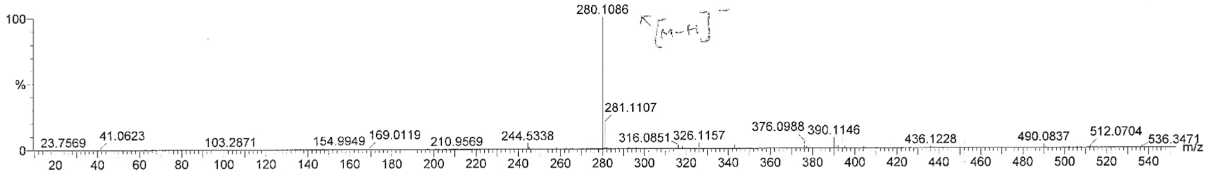

Minimum:				-100.0			
Maximum:		5.0	20.0	1000.0			
Mass	Calc. Mass	mDa	PPM	DBE	i-FIT	i-FIT (Norm)	Formula
280.1086	280.1086	0.0	0.0	11.5	125.3	0.0	C16 H14 N3 O2

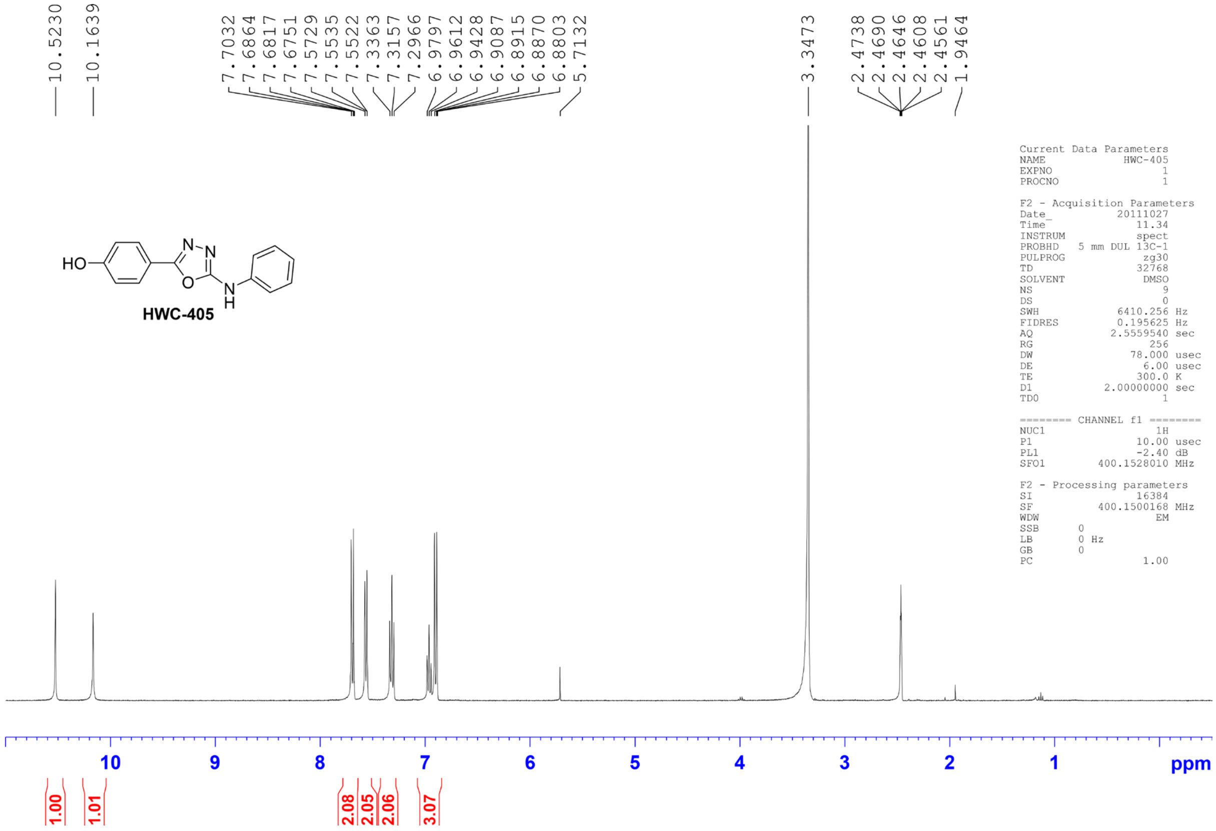

HWC-405

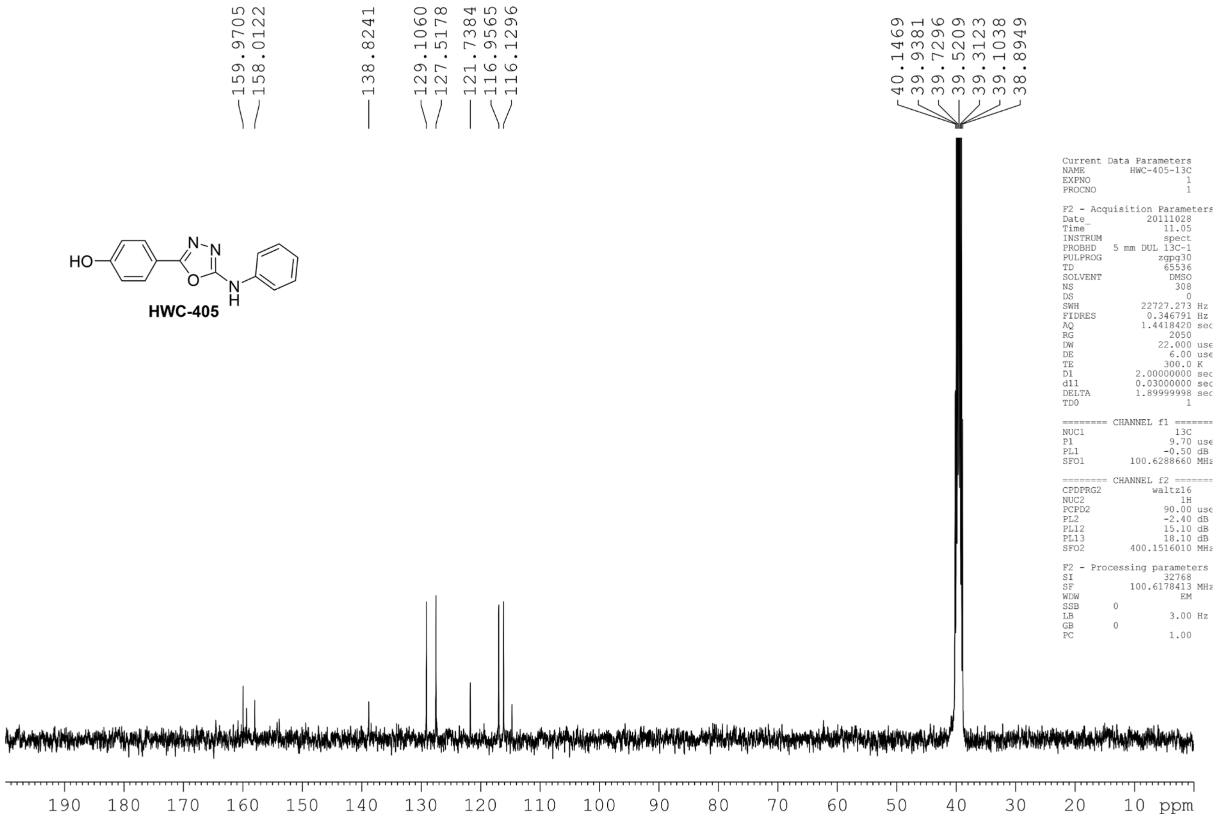

HWC-405 (HR-ESI neg)

Elemental Composition Report

Page 1

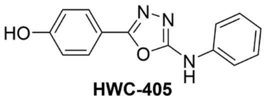

HWC-405

Single Mass Analysis

Tolerance = 100.0 PPM / DBE: min = -100.0, max = 1000.0
Element prediction: Off
Number of isotope peaks used for i-FIT = 2

Monoisotopic Mass, Even Electron Ions
15 formula(e) evaluated with 1 results within limits (all results (up to 1000) for each mass)
Elements Used:
C: 0-1000 H: 0-4000 N: 3-3 O: 2-2

HWC-405 KE267 05-Dec-2011 16:27:47
1202_HWC-405 6 (0.322) 1: TOF MS ES- 1.90e+003

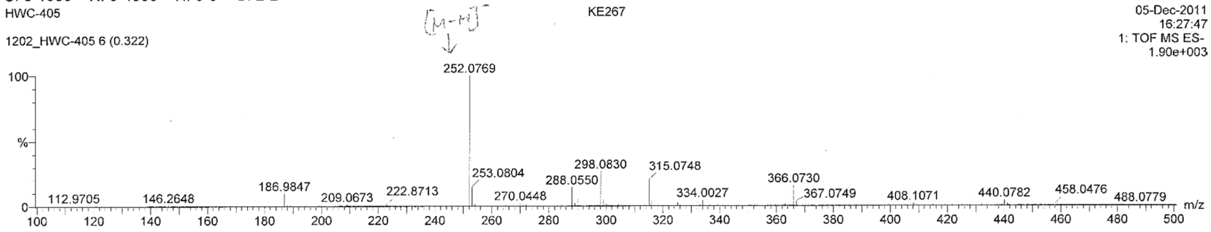

Minimum:				-100.0			
Maximum:		5.0	100.0	1000.0			
Mass	Calc. Mass	mDa	PPM	DBE	i-FIT	i-FIT (Norm)	Formula
252.0769	252.0773	-0.4	-1.6	11.5	62.1	0.0	C14 H10 N3 O2

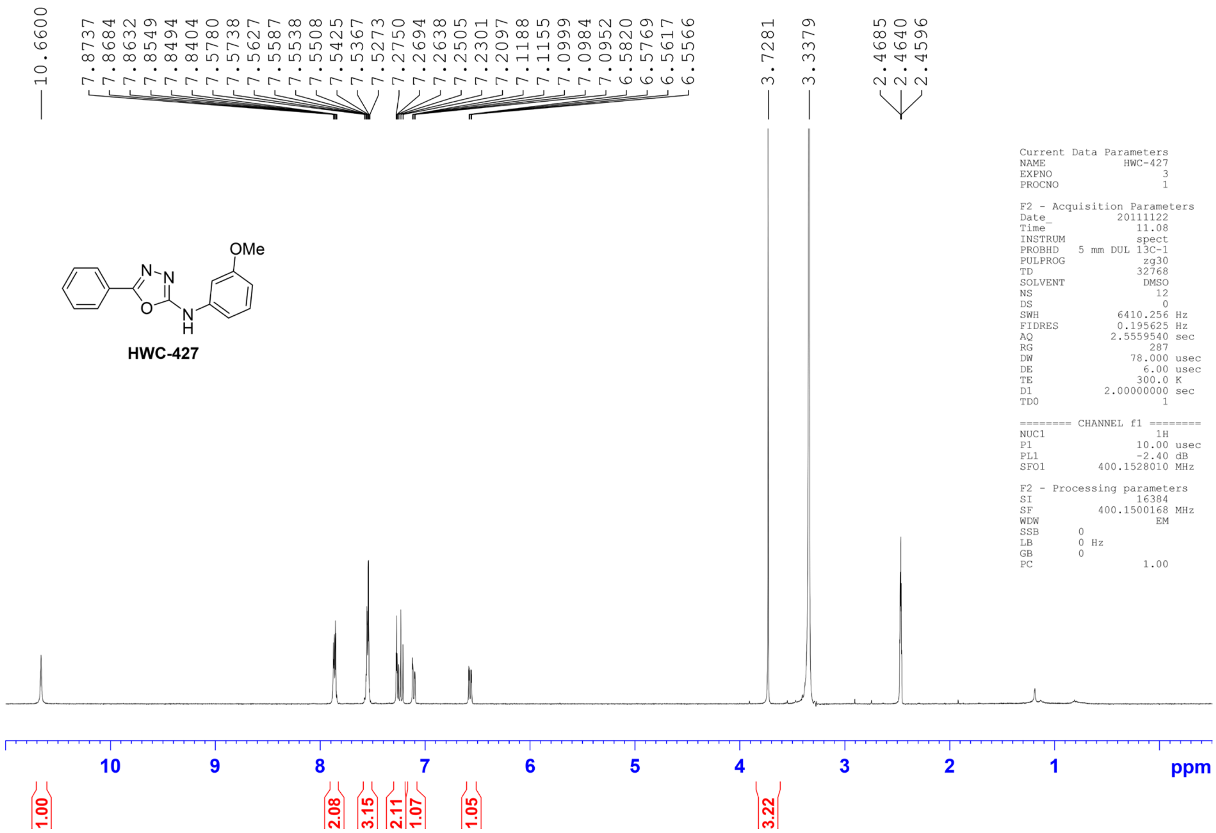
10.6600
7.8737 7.8684 7.8632 7.8549 7.8494 7.8404 7.5780 7.5738 7.5627 7.5587 7.5538 7.5508 7.5425 7.5367 7.5273 7.2750 7.2694 7.2638 7.2505 7.2301 7.2097 7.1188 7.1155 7.0999 7.0984 7.0952 6.5820 6.5769 6.5617 6.5566
3.7281
3.3379
2.4685 2.4640 2.4596
OMe
N-N
O
N
H
HWC-427
Current Data Parameters
NAME HWC-427
EXPNO 3
PROCNO 1
F2 - Acquisition Parameters
Date_ 20111122
Time 11.08
INSTRUM spect
PROBHD 5 mm DUL 13C-1
PULPROG zg30
TD 32768
SOLVENT DMSO
NS 12
DS 0
SWH 6410.256 Hz
FIDRES 0.195625 Hz
AQ 2.5559540 sec
RG 287
DW 78.000 usec
DE 6.00 usec
TE 300.0 K
D1 2.00000000 sec
TD0 1
======== CHANNEL f1 ========
NUC1 1H
P1 10.00 usec
PL1 -2.40 dB
SFO1 400.1528010 MHz
F2 - Processing parameters
SI 16384
SF 400.1500168 MHz
WDW EM
SSB 0
LB 0 Hz
GB 0
PC 1.00
10 9 8 7 6 5 4 3 2 1 ppm
1.00 2.08 3.15 2.11 1.07 1.05 3.22

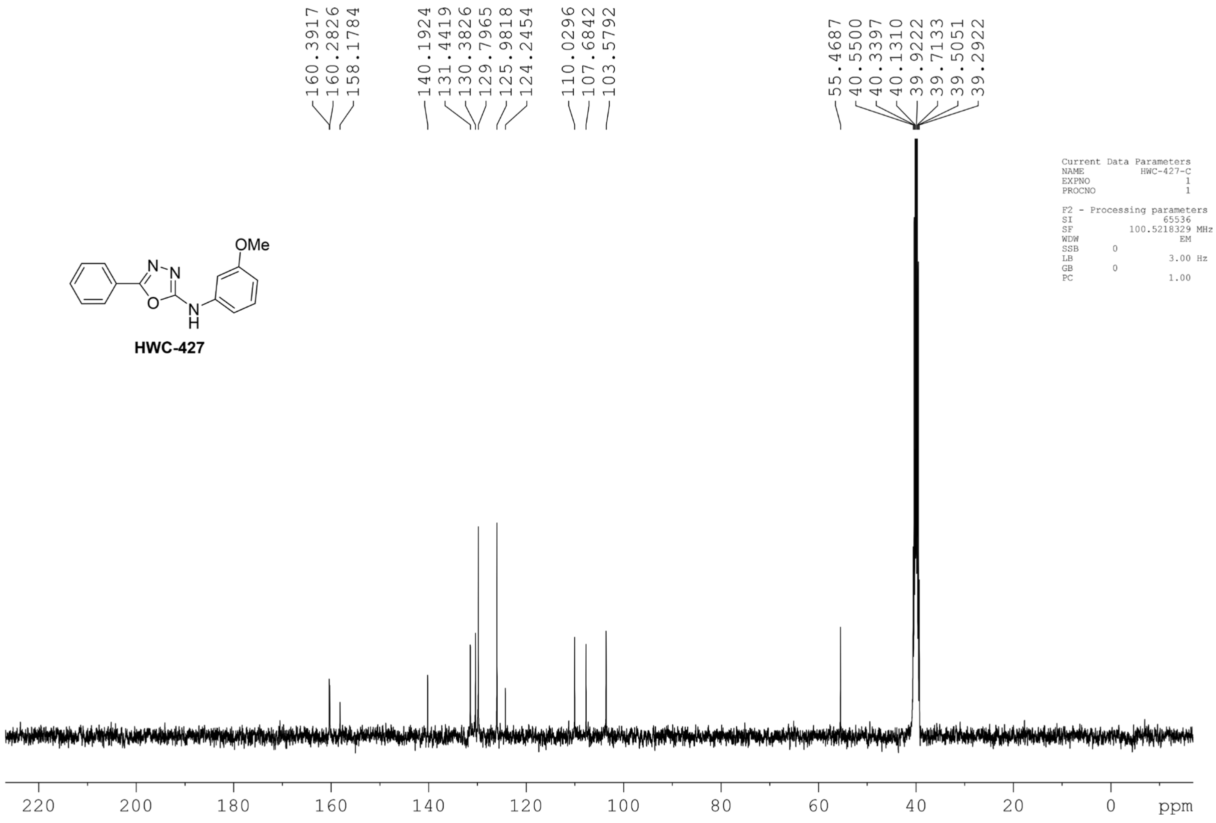
160.3917 160.2826 158.1784
140.1924 131.4419 130.3826 129.7965 125.9818 124.2454
110.0296 107.6842 103.5792
55.4687 40.5500 40.3397 40.1310 39.9222 39.7133 39.5051 39.2922
OMe
N-N
O
N
H
HWC-427
Current Data Parameters
NAME HWC-427-C
EXPNO 1
PROCNO 1
F2 - Processing parameters
SI 65536
SF 100.5218329 MHz
WDW EM
SSB 0
LB 3.00 Hz
GB 0
PC 1.00
220 200 180 160 140 120 100 80 60 40 20 0 ppm

HWC-427 (HR-ESI neg)

Elemental Composition Report

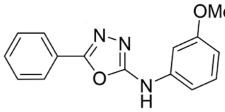

HWC-427

Single Mass Analysis
Tolerance = 100.0 PPM / DBE: min = -100.0, max = 1000.0
Element prediction: Off
Number of isotope peaks used for i-FIT = 2

Monoisotopic Mass, Even Electron Ions
17 formula(e) evaluated with 1 results within limits (all results (up to 1000) for each mass)
Elements Used:
C: 0-1000 H: 0-4000 N: 3-3 O: 2-2

HWC-427 KE267 05-Dec-2011 16:50:15
1202_HWC-427 19 (1.037) Cm (19-1) 1: TOF MS ES- 2.61e+004

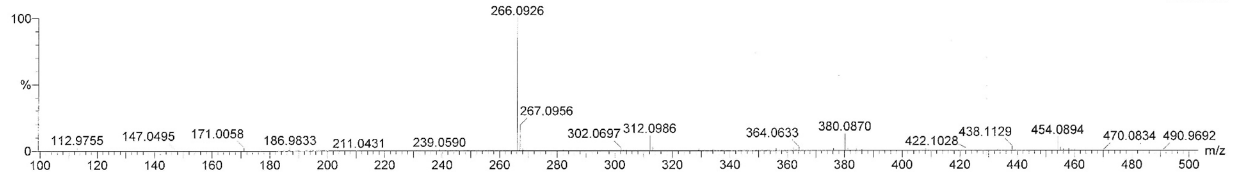

Minimum:				-100.0			
Maximum:		5.0	100.0	1000.0			
Mass	Calc. Mass	mDa	PPM	DBE	i-FIT	i-FIT (Norm)	Formula
266.0926	266.0930	-0.4	-1.5	11.5	183.2	0.0	C15 H12 N3 O2

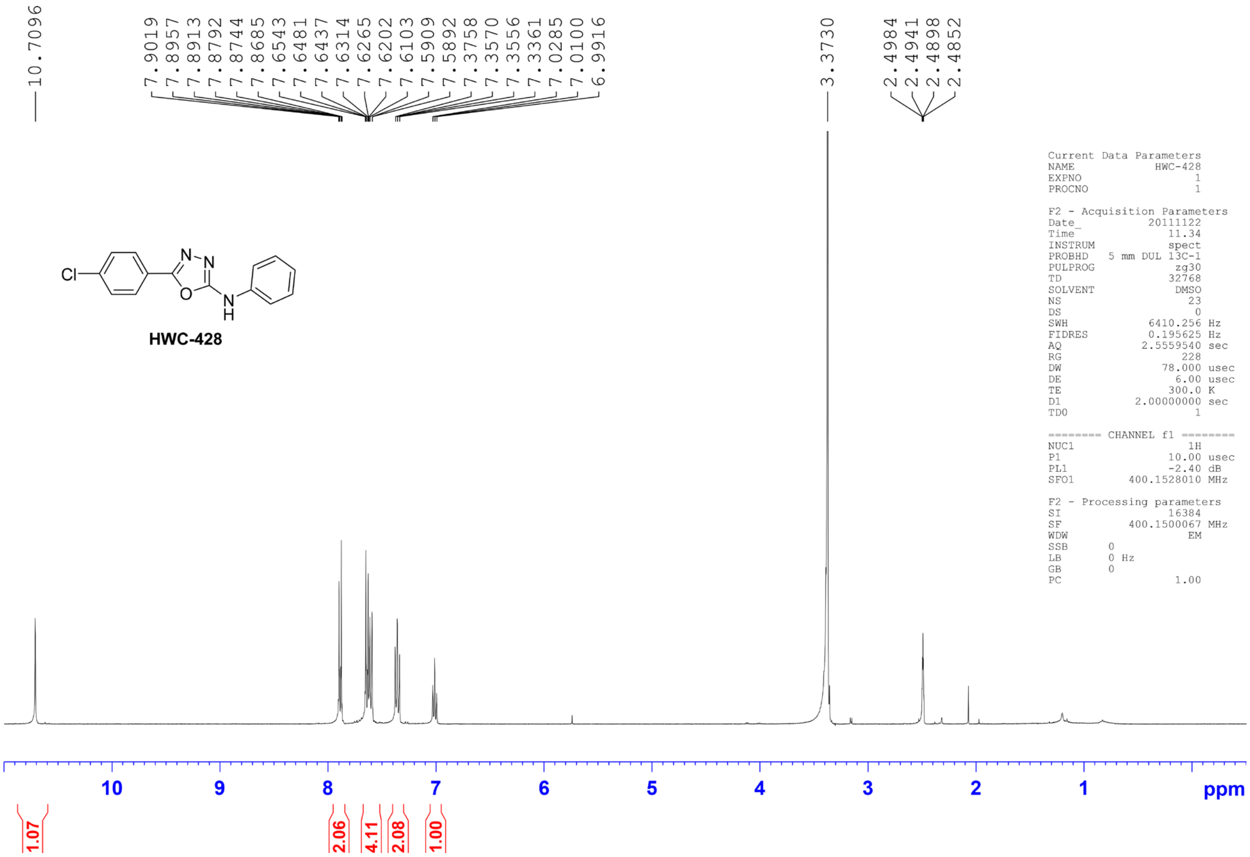

HWC-428

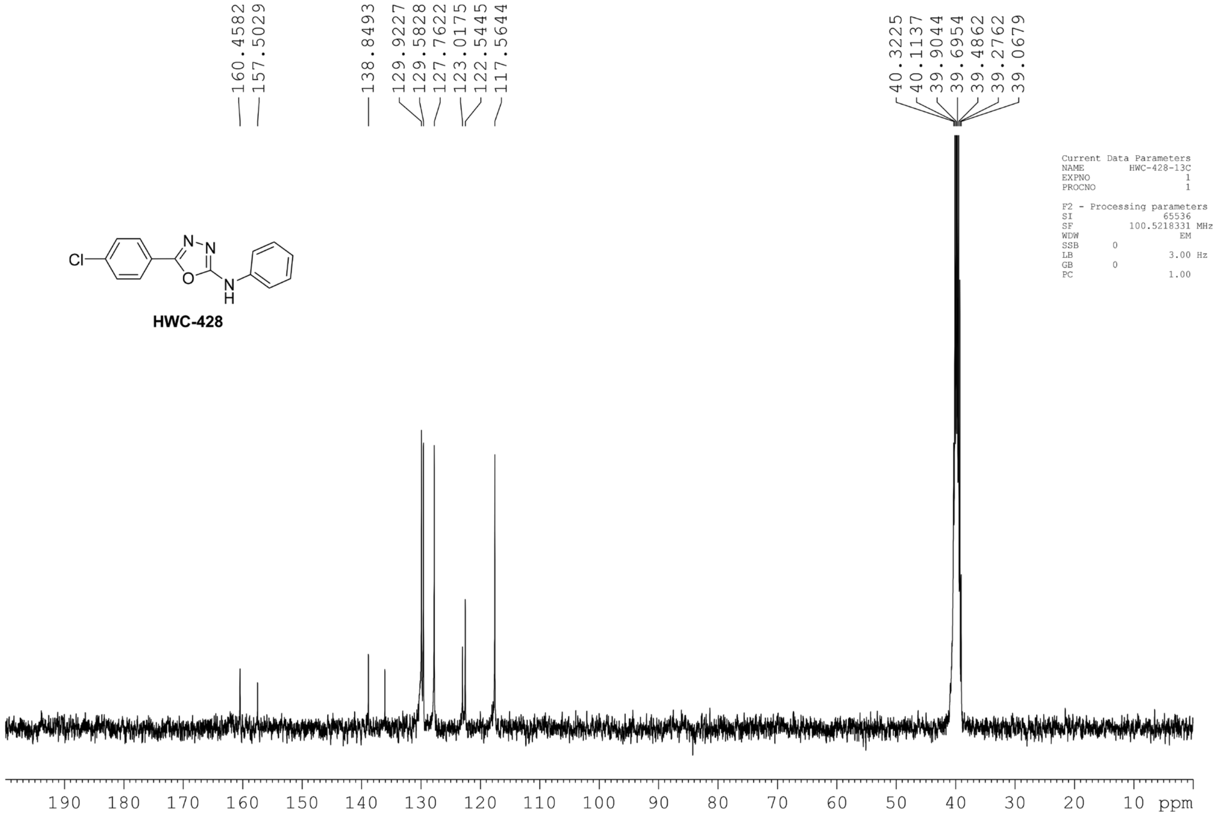

HWC-428 (CHR-ESI neg)

Elemental Composition Report

Page 1

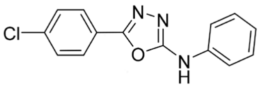

HWC-428

Single Mass Analysis
Tolerance = 100.0 PPM / DBE: min = -100.0, max = 1000.0
Element prediction: Off
Number of isotope peaks used for i-FIT = 2

Monoisotopic Mass, Even Electron Ions
15 formula(e) evaluated with 1 results within limits (all results (up to 1000) for each mass)
Elements Used:
C: 0-1000 H: 0-4000 N: 3-3 O: 1-1 Cl: 1-1
HWC-428

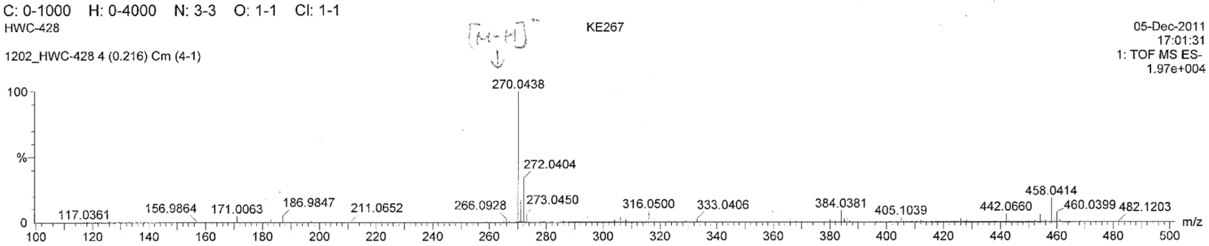

Minimum:				-100.0			
Maximum:		5.0	100.0	1000.0			
Mass	Calc. Mass	mDa	PPM	DBE	i-FIT	i-FIT (Norm)	Formula
270.0438	270.0434	0.4	1.5	11.5	217.7	0.0	C14 H9 N3 O Cl

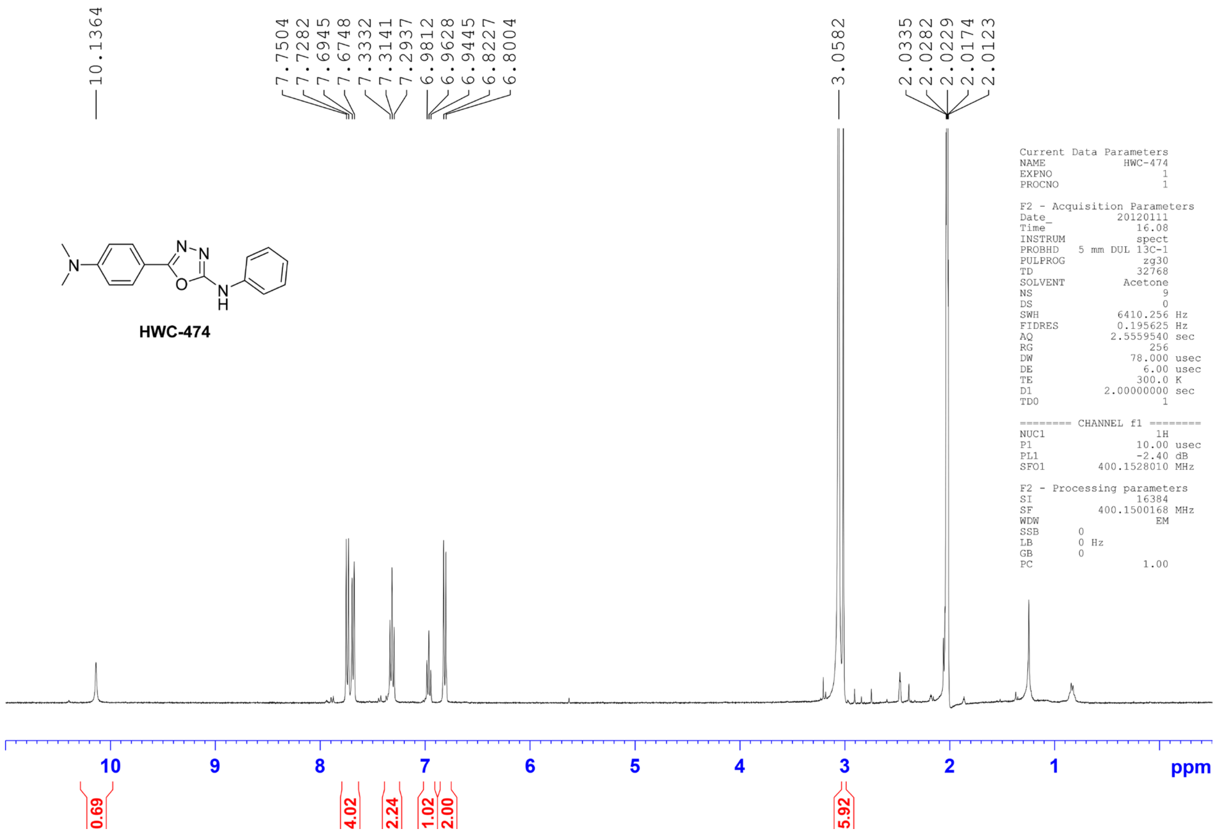
10.1364
7.7504
7.7282
7.6945
7.6748
7.3332
7.3141
7.2937
6.9812
6.9628
6.9445
6.8227
6.8004
3.0582
2.0335
2.0282
2.0229
2.0174
2.0123
HWC-474
Current Data Parameters
NAME HWC-474
EXPNO 1
PROCNO 1
F2 - Acquisition Parameters
Date_ 20120111
Time 16.08
INSTRUM spect
PROBHD 5 mm DUL 13C-1
PULPROG zg30
TD 32768
SOLVENT Acetone
NS 9
DS 0
SWH 6410.256 Hz
FIDRES 0.195625 Hz
AQ 2.5559540 sec
RG 256
DW 78.000 usec
DE 6.00 usec
TE 300.0 K
D1 2.00000000 sec
TD0 1
======== CHANNEL f1 ========
NUC1 1H
P1 10.00 usec
PL1 -2.40 dB
SFO1 400.1528010 MHz
F2 - Processing parameters
SI 16384
SF 400.1500168 MHz
WDW EM
SSB 0
LB 0 Hz
GB 0
PC 1.00
10
9
8
7
6
5
4
3
2
1
ppm
0.69
4.02
2.24
1.02
2.00
5.92

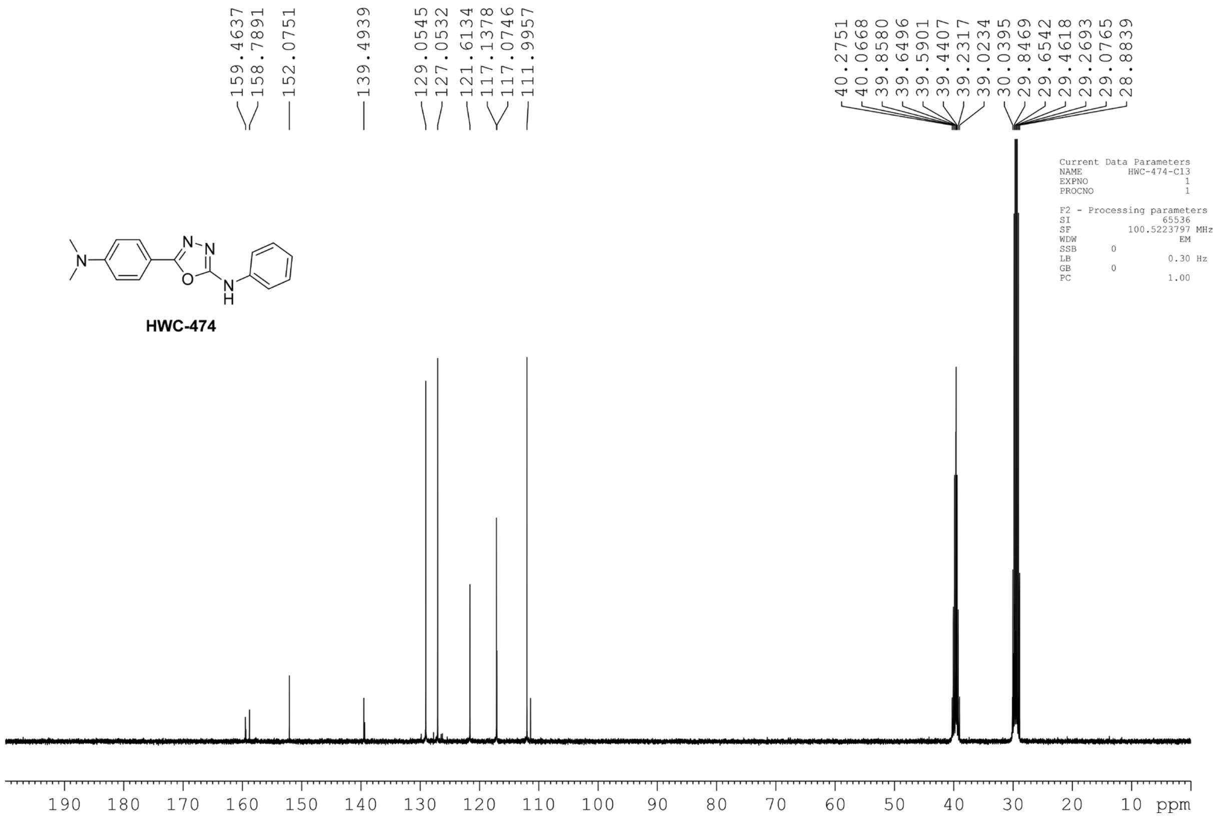
159.4637
158.7891
152.0751
139.4939
129.0545
127.0532
121.6134
117.1378
117.0746
111.9957
40.2751
40.0668
39.8580
39.6496
39.5901
39.4407
39.2317
39.0234
30.0395
29.8469
29.6542
29.4618
29.2693
29.0765
28.8839
HWC-474
Current Data Parameters
NAME HWC-474-C13
EXPNO 1
PROCNO 1
F2 - Processing parameters
SI 65536
SF 100.5223797 MHz
WDW EM
SSB 0
LB 0.30 Hz
GB 0
PC 1.00
190
180
170
160
150
140
130
120
110
100
90
80
70
60
50
40
30
20
10
ppm

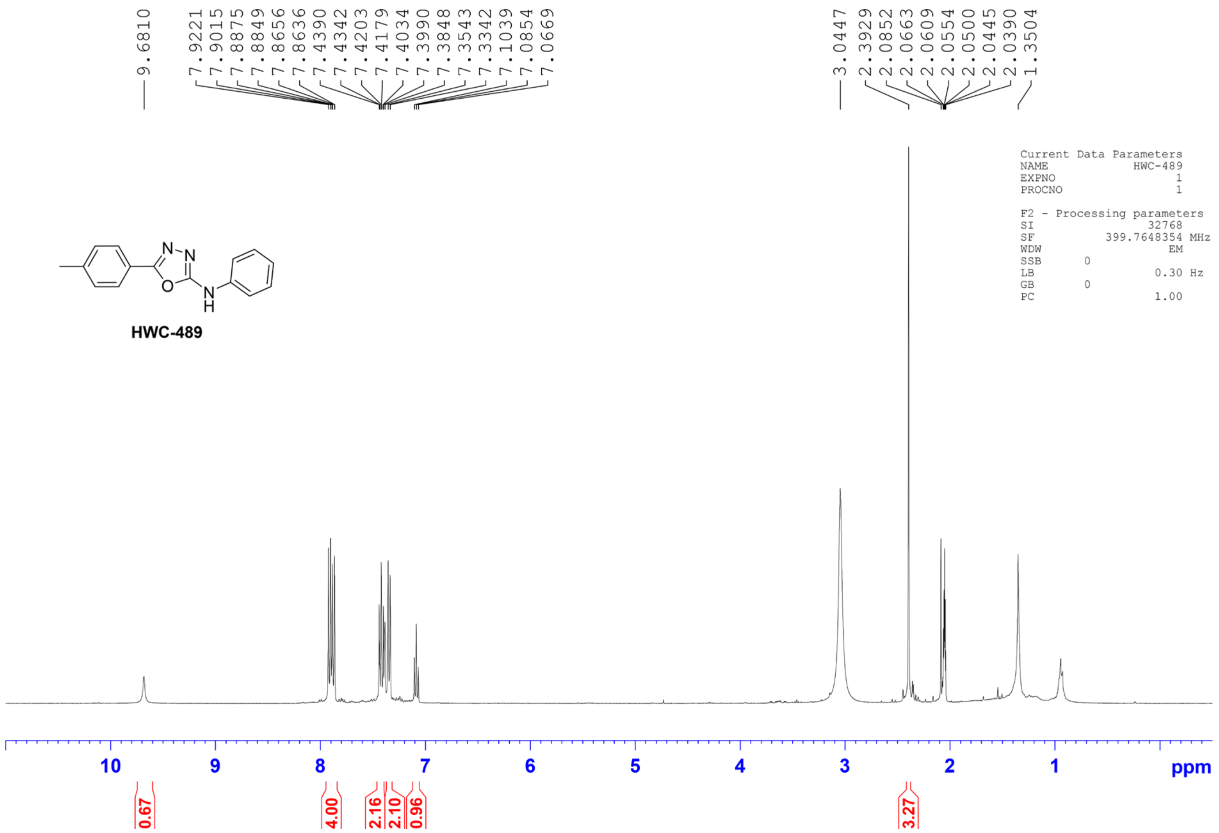
9.6810
7.9221
7.9015
7.8875
7.8849
7.8656
7.8636
7.4390
7.4342
7.4203
7.4179
7.4034
7.3990
7.3848
7.3543
7.3342
7.1039
7.0854
7.0669
3.0447
2.3929
2.0852
2.0663
2.0609
2.0554
2.0500
2.0445
2.0390
1.3504
Current Data Parameters
NAME HWC-489
EXPNO 1
PROCNO 1
F2 - Processing parameters
SI 32768
SF 399.7648354 MHz
WDW EM
SSB 0
LB 0.30 Hz
GB 0
PC 1.00
HWC-489
10 9 8 7 6 5 4 3 2 1 ppm
0.67
4.00
2.16
2.10
0.96
3.27

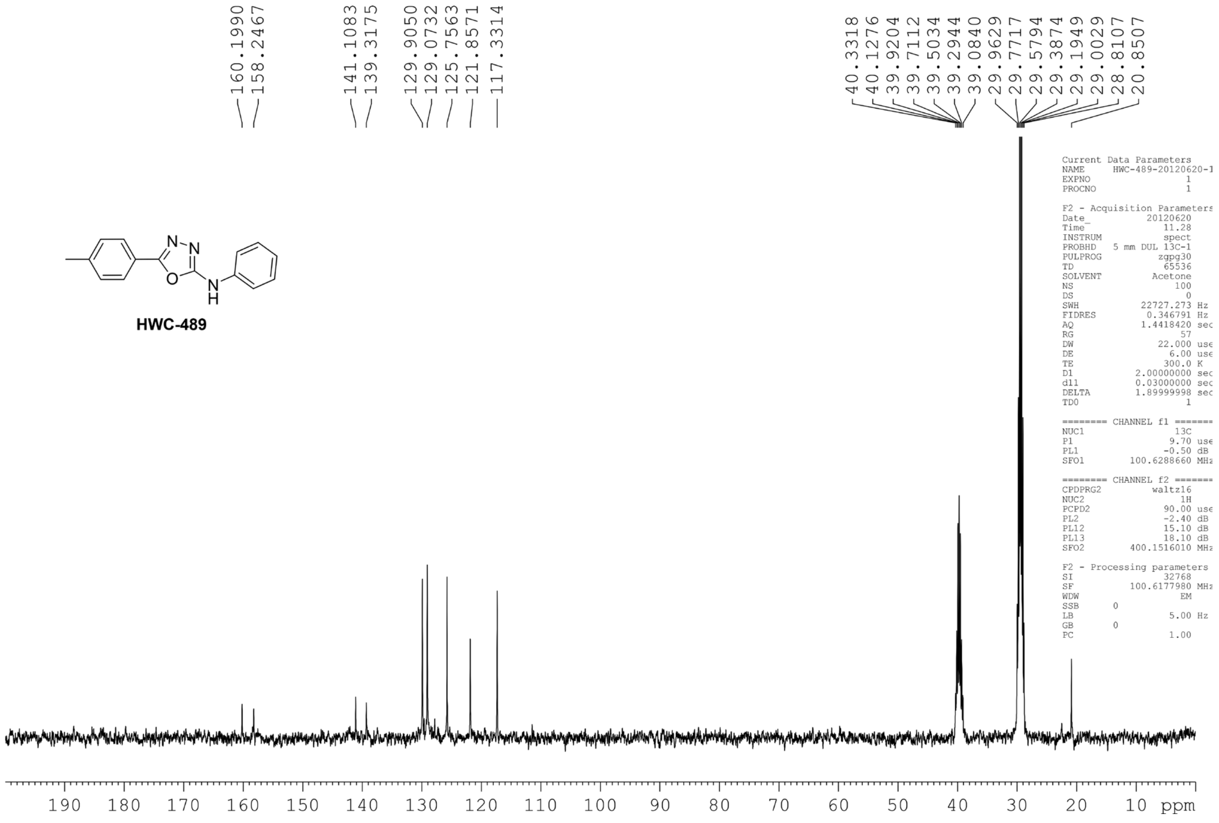
160.1990
158.2467
141.1083
139.3175
129.9050
129.0732
125.7563
121.8571
117.3314
40.3318
40.1276
39.9204
39.7112
39.5034
39.2944
39.0840
29.9629
29.7717
29.5794
29.3874
29.1949
29.0029
28.8107
20.8507
Current Data Parameters
NAME HWC-489-20120620-1
EXPNO 1
PROCNO 1
F2 - Acquisition Parameters
Date_ 20120620
Time 11.28
INSTRUM spect
PROBHD 5 mm DUL 13C-1
PULPROG zgpg30
TD 65536
SOLVENT Acetone
NS 100
DS 0
SWH 22727.273 Hz
FIDRES 0.346791 Hz
AQ 1.4418420 sec
RG 57
DW 22.000 usec
DE 6.00 usec
TE 300.0 K
D1 2.00000000 sec
d11 0.03000000 sec
DELTA 1.89999998 sec
TD0 1
======== CHANNEL f1 ========
NUC1 13C
P1 9.70 usec
PL1 -0.50 dB
SFO1 100.6288660 MHz
======== CHANNEL f2 ========
CPDPRG2 waltz16
NUC2 1H
PCPD2 90.00 usec
PL2 -2.40 dB
PL12 15.10 dB
PL13 18.10 dB
SFO2 400.1516010 MHz
F2 - Processing parameters
SI 32768
SF 100.6177980 MHz
WDW EM
SSB 0
LB 5.00 Hz
GB 0
PC 1.00
HWC-489
190 180 170 160 150 140 130 120 110 100 90 80 70 60 50 40 30 20 10 ppm

HWC-489 (HR-ESI neg)

Elemental Composition Report

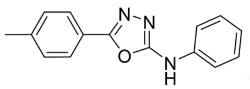

HWC-489

Page 1

Single Mass Analysis
Tolerance = 20.0 PPM / DBE: min = -100.0, max = 1000.0
Element prediction: Off
Number of isotope peaks used for i-FIT = 2

Monoisotopic Mass, Even Electron Ions
17 formula(e) evaluated with 1 results within limits (all results (up to 1000) for each mass)
Elements Used:
C: 0-4000 H: 0-4000 N: 3-3 O: 1-1

HWC-489 KE267 07-Mar-2012
12:33:52
0307_HWC-489 10 (1.013) Cm (10-2) 2: TOF MS ES-
3.58e+003

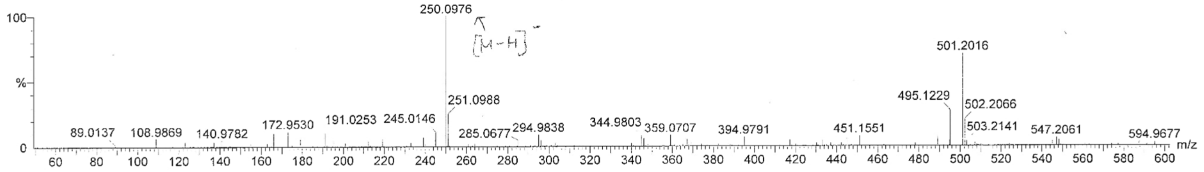

Minimum:				-100.0			
Maximum:		5.0	20.0	1000.0			
Mass	Calc. Mass	mDa	PPM	DBE	i-FIT	i-FIT (Norm)	Formula
250.0976	250.0980	-0.4	-1.6	11.5	57.3	0.0	C15 H12 N3 O

Supplementary Figure 1: Synthesis pathways of oxadiazole derivatives.
